# Human Umbilical Cord Plasma Metabolomics Uncover Potential Metabolites for Combating Aging

**DOI:** 10.1111/acel.70295

**Published:** 2025-11-26

**Authors:** JiaYu Liu, Shuai Jiang, YanYan Shen, RuiBo Wang, Zhi Jin, YanQing Cao, JinLiang Li, YanHong Liu, Qi Qi, Yue Guo, YunYing Wang, BoYang Xie, JunCheng Li, AiPing Cao, Yao Wang, ChunYan Yan, QiuYing Han, YingJie Zhu, Jing Peng, FangTing Dong, Xin Pan, XinHua He, Tao Zhou, AiLing Li, Kun He, Na Wang, WeiNa Zhang, Qing Xia

**Affiliations:** ^1^ Nanhu Laboratory State Key Laboratory of Biomedical Analysis (SKLBA, Formerly Known as National Center of Biomedical Analysis, NCBA) Beijing China; ^2^ State Key Laboratory of Toxicology and Medical Countermeasures Institute of Pharmacology and Toxicology Beijing China

**Keywords:** anti‐aging, human umbilical cord blood, lifespan extension, metabolites, metabolomics

## Abstract

Human umbilical cord blood (HUCB) exhibits distinct characteristics compared to adult blood, offering significant potential for medical applications, particularly in antiaging therapies. However, the metabolic profile of HUCB relative to adult blood remains poorly understood. Moreover, the specific metabolites within HUCB that confer antiaging properties have yet to be identified. Here, we conducted an untargeted metabolomic analysis comparing cord plasma and adult plasma. Our results reveal a unique metabolic landscape in cord plasma, characterized by significant differences in 662 out of 1092 total compounds and 43 out of 59 total human metabolic pathways. Notably, 211 abundant cord metabolites decline with age, involving key aging‐related processes, including inflammation, oxidative stress, energy and nutrition metabolism, proteostasis and DNA damage responses, implicating their potential role in counteracting aging. Importantly, a proof‐of‐concept experiment demonstrates that a formula containing five of these metabolites (carnosine, taurocholic acid, inosine, L‐Histidine and N‐acetylneuraminic acid) significantly extends both lifespan and healthspan in *C. elegans*. Collectively, our findings provide novel insights into the distinctive characteristics of the human cord plasma metabolome and identify promising metabolites with therapeutic potential for antiaging and other cord blood‐based medical applications.

## Graphical abstract

Untargeted metabolomics reveals a unique cord plasma metabolic landscape versus adult. Age‐declining cord‐specific enriched metabolites are identified as antiaging candidates, with a 5‐compound formula validated in proof‐of‐experiments to extend lifespan and healthspan in human cells and *C. elegans*.

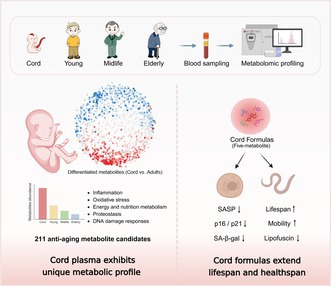

## 1 Background

Human umbilical cord blood (HUCB) displays distinct biochemical and cellular features compared to adult blood, harboring a unique repertoire of hematopoietic stem cells and specialized molecular entities. This distinctive composition underlies its established therapeutic utility across a spectrum of pathologies, including tissue damage, immune dysregulation, and neurological disorders (Ballen et al. 2013; Nagamura‐Inoue and Nagamura 2023). Recently, accumulating evidence has further emphasized its substantial promise for antiaging interventions (Bae et al. 2019; Castellano et al. 2017; Mei et al. 2021).

Parabiosis studies, where the circulatory systems of young and aged mice are surgically connected, have demonstrated that youthful blood can reverse age‐associated functional deterioration across diverse organ systems, including muscle, pancreas, liver, heart and brain (Ma et al. 2022; Palovics et al. 2022; Villeda et al. 2014). Notably, transfusion of human umbilical cord plasma, the youngest form of human plasma, to aged mice markedly enhances cognitive function (Castellano et al. 2017). Concomitantly, accumulating evidence indicates that young blood harbors intrinsic antiaging factors (Castellano et al. 2017; Kang and Yang 2020; Yates 2014). Such factors identified in young blood encompass both proteins and endogenous small‐molecule metabolites.

Metabolism constitutes a defining feature of life, with small‐molecule metabolites integral to maintaining systemic homeostasis. Beyond their canonical roles in substance conversion and energy transduction, these metabolites mediate critical noncanonical functions in signal transduction, orchestrating key roles in diverse physiological and pathological processes including cell proliferation, neural signaling, carcinogenesis, and neurodegeneration (Baker and Rutter 2023; Foh et al. 2022). Recent studies have elucidated that certain metabolites act as pivotal regulators of aging and age‐related pathologies (Chin et al. 2014; Eisenberg et al. 2016; Madeo et al. 2018; Mouchiroud et al. 2013; Naeini et al. 2023; Rajman et al. 2018; Rhoads and Anderson 2020; Singh et al. 2023; Zhang et al. 2016). For instance, alpha‐ketoglutarate (α‐KG), an intermediate of the tricarboxylic acid (TCA) cycle, extends lifespan in *C. elegans* and exerts antiaging effects in mice (Chin et al. 2014; Naeini et al. 2023; Rhoads and Anderson 2020). Similarly, precursors of Nicotinamide adenine dinucleotide (NAD), such as nicotinamide mononucleotide (NMN) and nicotinamide riboside (NR), have demonstrated efficacy in promoting longevity in nematodes, extending murine lifespan, and improving healthspan during aging (Mouchiroud et al. 2013; Rajman et al. 2018; H. Zhang et al. 2016). Additionally, spermidine, another endogenous metabolite, is recognized for its cardioprotective properties and capacity to extend mouse lifespan (Eisenberg et al. 2016; Madeo et al. 2018). Most recently, Singh et al. demonstrated that taurine supplementation enhances both healthspan and lifespan in mice (Singh et al. 2023). Moreover, given their natural occurrence, favorable safety profiles, and therapeutic potential, there is growing interest in utilizing endogenous metabolites to develop strategies for combating aging.

Accumulating evidence indicates that HUCB contains a rich repertoire of endogenous antiaging factors. For instance, the protein Tissue Inhibitor of Metalloproteinase 2 (TIMP2) has been identified as a key contributor to HUCB‐mediated alleviation of age‐related declines in hippocampal cognitive function in aged mice. However, the specific metabolites responsible for HUCBs antiaging activity remain to be defined. Adding to this limitation, while untargeted metabolomic profiling of adult blood has been extensively pursued, comprehensive investigations into umbilical cord plasma remain scarce (Cheng et al. 2015; Darst et al. 2019; Kaupper et al. 2023; Liang et al. 2020; Montoliu et al. 2014; Shen et al. 2020; Voerman et al. 2020). Notably, comparative metabolomic analyses that explicitly focus on the differential metabolites between umbilical cord blood and adult blood have yet to be reported.

In the present study, we performed an untargeted comparative metabolomic analysis of human neonatal cord plasma and adult plasma, aiming to delineate the unique metabolomic features of cord blood and identify health‐promoting metabolites, including those with antiaging potential. Our analyses revealed a distinct metabolic signature in cord plasma, with 662 of 1092 detected compounds exhibiting significant abundance differences relative to adult plasma. Notably, 211 metabolites enriched in cord blood, whose levels decline with age, are associated with key aging‐related biological processes, implicating their potential role in counteracting aging. Importantly, the proof‐of‐concept experiments demonstrated that formulas containing five such cord‐enriched metabolites extended lifespan and improved fitness in aged *C. elegans*. Collectively, this study uncovers the unique metabolic landscape of human umbilical cord plasma, identifying promising metabolites with potential for antiaging interventions and other cord blood‐based medical applications.

## 2 Results

### 2.1 Study Population

A total of 330 participants were enrolled in this study, comprising 60 mother‐offspring pairs and 270 healthy adults. None of the infants suffered from any birth defects. Maternal eligibility was defined as age < 35 years at enrollment, with exclusion criteria comprising hypertension, gestational diabetes mellitus (GDM), and other pregnancy‐related complications associated with adverse offspring outcomes. Adult participants were required to be free of metabolic comorbidities (hypertension, type 2 diabetes, dyslipidemia [hypertriglyceridemia, elevated total cholesterol], and obesity [Body Mass Index (BMI ≥ 30 kg/m^2^)]) and confirmed to be in good general health via medical history review.

**FIGURE 1:**
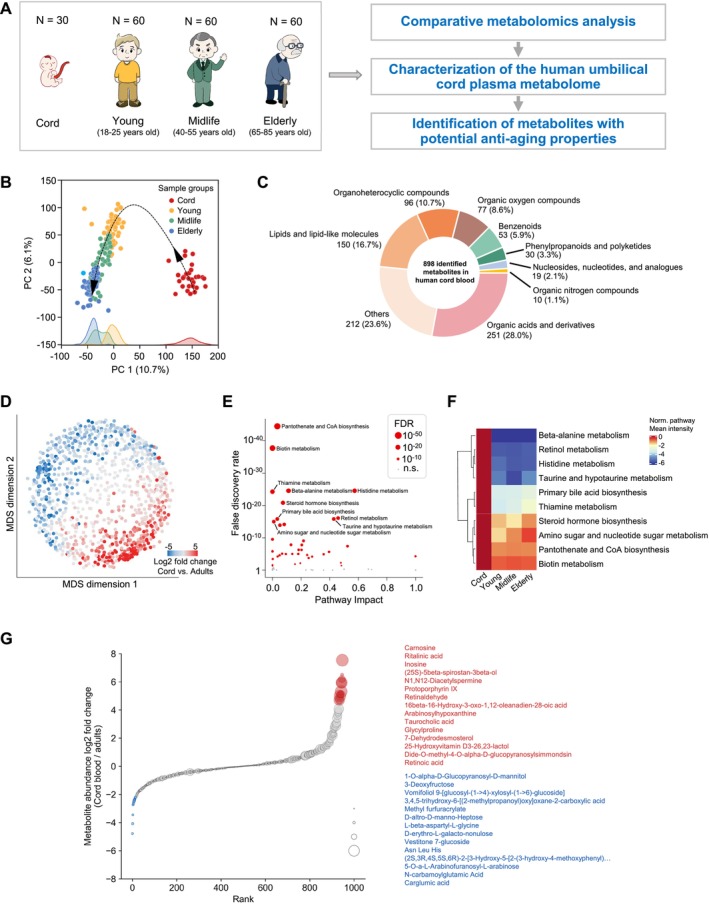
Comparison of metabolomic profiles between human umbilical cord plasma and adult plasma. (A) Experimental and data analysis scheme. (B) Scatterplot showing dimensional reduction of all samples based on all LC–MS features using PCA. The fill color of the scatters denotes the age groups, with a histogram of each group shown at the bottom. (C) Pie chart showing the annotations of all 898 identified metabolites in human cord plasma. (D) Scatterplot showing the 2‐dimensional embedding of all metabolites identified with high confidence using MDS. The fill color of the scatters denotes the log2 fold change of metabolite LS‐MS peak intensity in adult groups and in the cord blood group. (E) Pathway enrichment analysis showing differing pathways in adults compared with cord blood with FDR < 0.05. Red circles denote significant differing pathways and gray dots indicate no significant change in the metabolite pathways. The size of each dot represents the statistical significance. (F) Heatmap showing the mean abundance of metabolites in the top 10 differing metabolite pathways relative to the abundance level in cord plasma. (G) Plot showing the top 15 increased (red) and decreased (blue) metabolites in adults compared to cord blood. The size of each dot represents the statistical significance of the abundance difference between adult groups and the cord blood group.

Plasma specimens (*n* = 330) were balanced for sex distribution and allocated into two independent cohorts. For untargeted metabolomic profiling (Cohort 1, *n* = 210), the sample set included: umbilical cord plasma from term neonates (*n* = 30); plasma from young adults (18–25 years, *n* = 60); middle‐aged individuals (40–55 years, *n* = 60); and elderly individuals (65–85 years, *n* = 60) (Figure 1A). For validation analyses (Cohort 2, *n* = 120), matched sub cohorts were generated with equal group sizes and maintained a 1:1 sex ratio: umbilical cord plasma (*n* = 30); young adults (18–25 years, *n* = 30); middle‐aged individuals (40–55 years, *n* = 30); and elderly individuals (65–86 years, *n* = 30). Demographic and clinical characteristics of all participants are summarized in Table S1 (maternity/infants) and Table S2 (adults). The study protocol was approved by the Institutional Review Board of the Academy of Military Medical Science, Beijing, China (approval number: AF/SC‐08/02.250).

### 2.2 Metabolomic Profiling of Human Umbilical Cord and Adult Plasma

To characterize the human umbilical cord plasma metabolome and identify metabolites intrinsic to umbilical cord blood, especially with potential antiaging properties, we conducted a comprehensive nontargeted metabolomic study on umbilical cord plasma from neonates (*n* = 30), plasma from young adults (*n* = 60; aged 18–25 years), middle‐aged individuals (*n* = 60; aged 40–55 years), and elderly individuals (*n* = 60; aged 65–85 years) (Figure 1A).

By employing ultraperformance liquid chromatography‐electrospray ionization tandem mass spectrometry (UPLC‐ESI‐MS/MS), we conducted a comprehensive profiling of cord plasma metabolites and compared these profiles with those of adult plasma samples. To increase the coverage of metabolites, we utilized two complementary columns, reversed‐phase liquid chromatography (RPLC) and hydrophilic interaction liquid chromatography (HILIC), for each sample. This enabled us to analyze the sample using four modes: RPLC‐POS (positive ion mode), RPLC‐NEG (negative ion mode), HILIC‐POS (positive ion mode) and HILIC‐NEG (negative ion mode). To ensure the reliability of results, we conducted three technical replicates (UPLC–MS runs) for each analysis. After quality control, data filtering, and normalization, a total of 59,690 features (retention time with exact m/z) were identified across 208 samples, following the removal of 2 outlier samples with low metabolite detection. We performed principal component analysis (PCA) on these samples using all identified features. Samples of each age group tend to form a dense cluster while different age groups are predominantly separated. A trajectory along clusters with increasing age was observed (Figure 1B). The metabolic differences between cord plasma and adult groups, including young, middle‐aged and elderly, are more significant than those between the three adult groups, highlighting the distinct features of the cord plasma metabolome. For compound identification, we conducted searches in two extensively utilized compound databases: the Human Metabolome Database (HMDB) and METLIN. A total of 1092 compounds were confidently mapped to LC–MS features. Notably, the abundance of 662 of these compounds exhibited significant differences between cord plasma and adult samples, accounting for over 60% of the total compounds (Table S3, *Q* value < 0.05).

To refine our analysis of neonate‐specific metabolic features, we excluded 167 compounds in the following analyses. This exclusion comprised exogenous substances (e.g., lidocaine and penicillin G) and steroid hormone metabolites (e.g., corticosteroids, estrogenic steroids, androgenic steroids, and pregnenolone steroids), which are likely influenced by maternal factors during pregnancy and childbirth, as indicated by an asterisk (*) in Table S3. Among the remaining 925 metabolites, 898 compounds were detected in cord plasma, constituting the majority of the metabolites identified overall. We categorized the 898 compounds into nine groups according to their chemical taxonomy (Figure 1C, Table S4). In order to generate a comprehensive overview of the plasma metabolite differences between the cord plasma and adult groups, we employed multidimensional scaling (MDS) to project the 925 metabolites onto a two‐dimensional space. This approach allows for the visualization of compounds that exhibit similar abundance distribution across all samples, thereby positioning them in close proximity to one another. This map of human plasma metabolites further illustrates significant metabolic differences between cord and adult groups, with numerous metabolites exhibiting differing abundance levels (Figure 1D).

### 2.3 Metabolites with Distinct Differences Between Cord and Adult Plasma

To further investigate the differences between cord plasma and adult plasma metabolomes, we conducted an analysis of the global pathway changes for all annotated compounds using MetaboAnalyst (version 5.0). Out of the 59 Kyoto Encyclopedia of Genes and Genomes (KEGG) pathways that were mapped, 43 pathways exhibited significant changes (72.8%, adjusted FDR < 0.05, global test), indicating extensive differences in metabolic pathways between the cord and adult groups (Figure 1E, Table S5). It is noteworthy that all top 10 differing pathways, as shown in Figure 1F, exhibited higher metabolite abundance in cord plasma compared to adult plasma. These findings underscore a distinct metabolic profile in cord plasma that differs significantly from that of adult plasma.

To identify the metabolites that exhibited the most notable differences between cord blood and adult blood, we established a threshold of a 4‐fold difference in metabolite abundance between cord and adult plasma, considering the three adult groups as a collective entity. Applying this criterion, we identified a total of 56 metabolites with significantly differing abundance levels. Among these metabolites, the majority (42 out of 56) displayed higher abundance in cord blood, while 14 were found to be more abundant in adult blood (Table S6). Figure 1G graphically depicts the top 15 upregulated and 14 downregulated metabolites in cord plasma.

**FIGURE 2:**
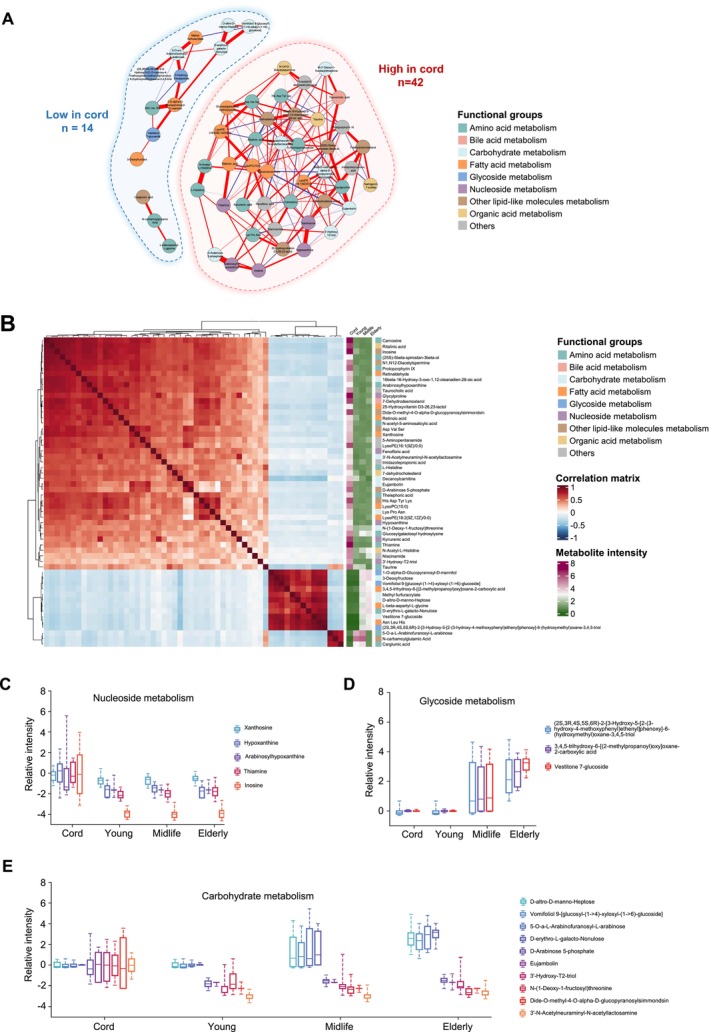
Significantly different metabolites between cord and adult plasma. (A) Regularized partial correlation network of top differing metabolites in adult plasma compared to cord blood. Each node in the network represents a metabolite and its color represents its biochemical annotation. Widths of the edges represent the strength of the partial correlation coefficient between two metabolites. (B) Heatmap showing detailed abundance of all top differing metabolites in all datasets, where each row corresponds to a metabolite and each column corresponds to a sample. Sample groups are marked at the bottom of the heatmap. (C–E) Boxplots showing the relative abundance level of metabolites annotated as nucleosides (C), glycosides (D) and carbohydrate (E) metabolism at each age group relative to cord blood.

To identify the functional groups of metabolites that underwent the most significant changes between cord and adult plasma, a correlation network was constructed using the top 56 differing metabolites. Based on known chemical and functional information, these metabolites were classified into 9 distinct functional groups. Notably, metabolites within the same functional group tended to cluster together on the correlation network (Figure 2A, Table S6). Importantly, metabolites that increased or decreased in cord blood formed distinct clusters. Specifically, the cord blood enriched metabolites formed a larger cluster, encompassing 8 out of 9 functional groups. Within this cluster, the bile acid metabolism and nucleosides metabolism groups exclusively resided, while the glycoside metabolism group was solely represented in the adult blood enriched cluster. Additionally, the majority of metabolites belonging to the amino acid metabolism, organic acid metabolism, and other lipid‐like molecules metabolism groups were present in the cord blood enriched cluster. The correlation matrix and mean metabolite abundances across groups are visualized in Figure 2B, along with the names and relative abundances of individual metabolites. The name and relative abundance of each metabolite are also listed. As depicted in Figure 2C,D, all five metabolites falling under the category of “nucleoside metabolism” exhibited a greater abundance in cord blood, whereas all three metabolites classified as part of “glycoside metabolism” displayed a higher prevalence in adults. The ten metabolites grouped under “carbohydrate metabolism” showed varying abundance patterns (Figure 2E). Collectively, our findings highlight significant differences between the cord plasma metabolome and that of adult plasma, with numerous metabolites displaying differing abundance levels and extensive metabolic pathway modifications. These results provide valuable insights into the metabolic changes occurring during the transition from fetal to adult life. The high‐abundance metabolites characteristic of cord plasma may include compounds with potential applications in regenerative medicine, immune regulation or antiaging therapeutics.

### 2.4 Identification and Functional Analysis of Aging‐Related Metabolites in Cord Blood

**FIGURE 3:**
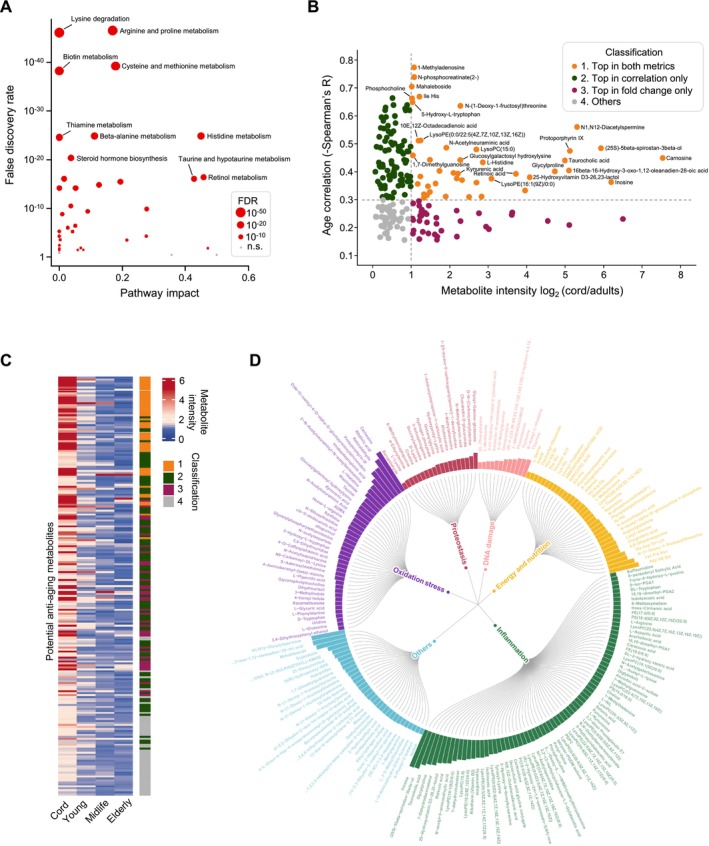
Identification and functional analysis of potential antiaging metabolites enriched in cord blood plasma. (A) Pathway enrichment analysis showing differing pathways in different age groups with FDR < 0.05. Red circles denote significant differing pathways and gray dots indicate no significant change in the metabolite pathway. The size of each dot represents the statistical significance. (B) Scatter plot showing two metrics used to identify potential antiaging metabolites. Each dot signifies a metabolite, with the color of the dot representing its associated group. (C) Heatmap showing the mean normalized abundances of all potential antiaging in each age group. (D) Circos‐like diagram showing the functional groups of all potential antiaging metabolites. The height of each bar represents the normalized metabolite abundance. The color of each bar represents the functional group of each metabolite.

Recent studies suggest that umbilical cord blood has antiaging properties. Based on this hypothesis, we sought to identify metabolite candidates in cord blood that exhibit antiaging properties, characterized by significantly higher abundance in cord blood and a declining trend with age progression. To achieve this, we employed a targeted identification approach to pinpoint these potential metabolites. Initially, we calculated the correlation between metabolite abundances and the age of the samples. We selected metabolites that displayed a negative correlation with age, applying a false discovery rate (FDR) cutoff of < 0.05. For these metabolites, we performed statistical tests to compare their abundance changes between cord blood and adult blood, filtering out those that did not show a significant decrease in adult blood, with the same FDR cutoff of < 0.05. Ultimately, 211 metabolites passed our two‐step filtering process and were identified as potential antiaging candidates (Table S7). To further explore the functionality of these metabolites, we performed an additional analysis using KEGG pathways. This analysis identified 34 distinct pathways with significant functional enrichment. Notably, the most prominent pathways were related to amino acid metabolism, including lysine degradation, arginine and proline metabolism, cysteine and methionine metabolism, thiamine metabolism, beta‐alanine metabolism and histidine metabolism (Figure 3A). Detailed results are provided in Table S8, highlighting the potential importance of these specific pathways.

To further classify these antiaging metabolite candidates, we grouped them based on two criteria: (i) the fold change in abundance between cord and adult plasma, and (ii) the inverse correlation between their abundances and age. The cut‐off values for these criteria were set at a fold change of 2 and a correlation coefficient of 0.3, respectively (Figure 3B, Table S7). To comprehensively evaluate the antiaging potential of all 211 aging‐related metabolites, we ranked each metabolite according to both criteria and integrated these ranks to develop a composite metric for antiaging potential (Figure 3C). Our analysis revealed that the top‐ranked metabolites were predominantly from group 1, which exhibited both high enrichment in cord plasma and a strong inverse correlation with age. Group 1, comprising 42 metabolites, consistently ranked highest on both metrics and thus was considered the most promising for antiaging applications. Applying contrast criteria, we also identified a number of metabolites potentially involved in promoting aging. These metabolites were found to be significantly less abundant in cord blood compared to adult blood and exhibited an increase in abundance with age. A total of 101 metabolites with these characteristics were identified (Figure S1A, Table S9). KEGG pathway analysis revealed 16 distinct pathways with significant functional enrichment, including glycerophospholipid metabolism and the citrate cycle (Figure S1B, Table S10). Future work will focus on experimentally testing these candidate metabolites to determine their association with aging and to identify those that may modulate the aging process. Based on known functional annotations, we further categorized the 211 antiaging metabolite candidates into six functional groups (Figure 3D, Table S7). The three largest functional groups among these metabolites were “inflammation”, “oxidative stress”, and “energy and nutrition”. Moreover, we identified 19 metabolites involved in proteostasis and 12 metabolites associated with DNA damage processes. These five functional groups correspond to five well‐established biological processes implicated in aging, providing further support for the antiaging potential of these 211 metabolites.

To validate the age‐related metabolite patterns identified in our initial untargeted analysis, we analyzed an independent cohort of 120 samples (30 per group: cord blood, young adults [18–25 years], middle‐aged [40–55 years], and elderly [65–85 years]), with strict matching of age and sex distributions to the discovery cohort to ensure comparability (Tables S1 and S2). Using LC–MS with calibration curves constructed from authentic compound standards, we quantified three metabolites (carnosine, taurocholic acid, and inosine) that emerged as candidates in group 1 from our untargeted screening.

Consistent with the untargeted metabolomic findings, all three metabolites exhibited significantly higher levels in cord plasma relative to all adult groups (Figure S2A–C). Taurocholic acid displayed a striking progressive age‐related decline across the lifespan. Its abundance peaked in cord blood, decreased gradually in young adults, declined further in middle‐aged individuals, and remained low in the elderly—directly confirming the age‐associated trend observed in the initial untargeted analysis (Figure S2B and Table S7). For carnosine and inosine, cord blood levels were markedly elevated compared to all adult subgroups. While these two metabolites did not show further declines across young, middle‐aged, and elderly adults, all adult subgroups maintained levels significantly lower than those in cord blood—consistent with the core pattern of adult‐stage reduction identified in our initial untargeted metabolomic analysis (Figure S2A,C and Table S7).

To further strengthen this validation, we extended our analysis to six additional candidate metabolites in a subcohort of 40 samples (10 per group: cord blood, young adults, middle‐aged, and elderly), with age and sex distributions matched to our main cohort. As visualized in Figure S2D–I, these six metabolites consistently showed peak abundance in cord plasma, with reduced levels across all adult groups—closely recapitulating the core trend from our initial findings (Table S7). Although certain metabolites (e.g., hypoxanthine and 1‐methyladenosine) maintained relatively stable levels among middle‐aged and elderly groups, all adult subgroups exhibited significantly lower abundances compared to cord blood, reinforcing the robustness of the untargeted metabolomic observation that these metabolites are enriched in cord blood and diminished in adulthood.

### 2.5 Metabolites in Cord Blood Attenuating Stress‐Induced Senescence in Human Embryonic Lung Fibroblasts

**FIGURE 4:**
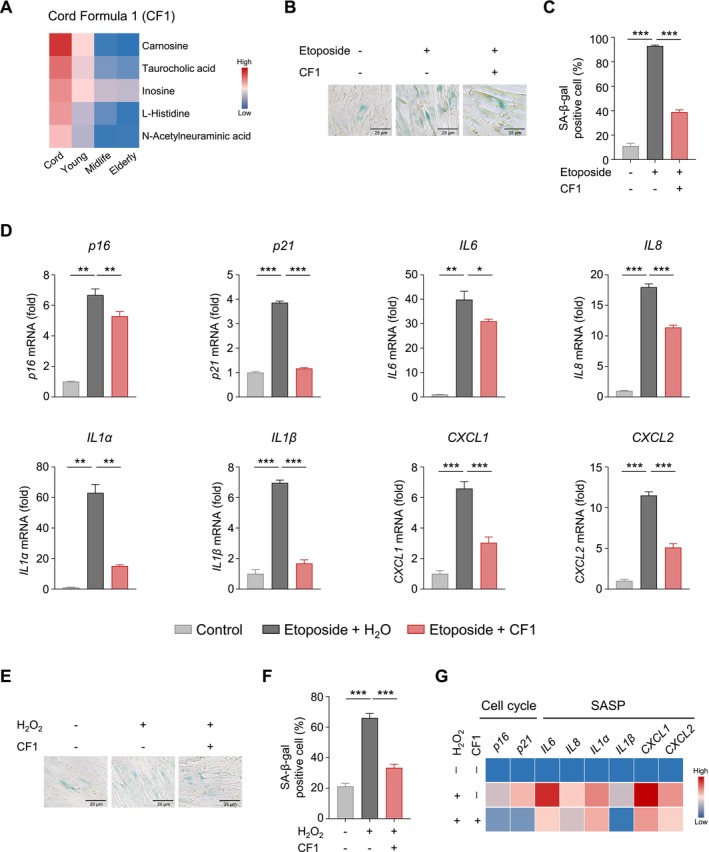
Attenuation of stress‐induced senescence in human embryonic lung fibroblasts by CF1. (A) Heatmap displaying the mean normalized abundances of metabolites composing CF1. (B‐C) Representative images (B) and statistical analysis (C) of SA‐β‐gal staining in MRC‐5 cells pretreated with or without CF1 and then exposed to 10 μM etoposide for 24 h, followed by washing and continuous culture in complete medium for 5 days. Scale bars, 25 μm. More than 500 cells were quantified in each group. (D) qPCR analysis of senescence‐associated genes in MRC‐5 cells treated as described in B. (E‐F) Representative images (E) and quantitative analysis (F) of SA‐β‐gal staining in MRC‐5 cells pretreated with or without CF1, exposed to 200 μM H₂O₂ for 1 h, and then cultured for 3 days. Scale bars, 25 μm. More than 500 cells were quantified in each group. (G) Heatmap analysis of senescence‐associated genes in MRC‐5 cells treated as described in E. *p < 0.05, **p < 0.01, ***p < 0.001. by unpaired *t* test. Values are mean ± SD.

To validate the antiaging potential of the candidate metabolites identified in untargeted metabolomic analysis, we selected five candidates to develop a formula, designated as the Cord‐Formula 1 (CF1), and conducted a proof‐of‐concept experiment. The five metabolites—carnosine, taurocholic acid, inosine, L‐histidine and N‐acetylneuraminic acid—were ranked among top in Group 1 (Figure 3B). These metabolites exhibited significant enrichment in umbilical cord plasma compared to adult blood and demonstrated a marked decline with aging (Figure 4A).

The identifications of all five metabolites were confirmed using chemical standards (Figure S3A–E). To elucidate the mechanisms underlying the enrichment of these metabolites in umbilical cord blood, we examined the transcriptional profiles of their cognate synthases or hydrolases across three cohorts: umbilical cord blood (*n* = 6), young adults (18–22 years, *n* = 5; 2 male/3 female), and elderly individuals (62–74 years, *n* = 5; 2 males/3 females). This analysis utilized two publicly available peripheral blood mononuclear cell (PBMC) RNA sequencing datasets: (1) umbilical cord blood PBMC RNA‐seq (GEO accession: GSE136849) and (2) adult PBMC RNA‐seq (GEO accession: GSE153100). All participants were free of medical conditions that could interfere with this study, with their demographic and clinical characteristics comprehensively documented in the original source publications (Chen et al. 2020; Wahl et al. 2021). RNA‐seq analysis revealed that key synthases—including CARNS1, SIAE, and NT5C—displayed significantly upregulated expression in cord blood versus adult blood. These upregulated expression levels positively correlated with elevated abundances of carnosine, N‐acetylneuraminic acid, and inosine (Figure S3F–H). Conversely, the hydrolase METTL6 exhibited downregulated expression in cord blood, which may explain the higher L‐histidine levels observed in cord blood versus adult blood (Figure S3I). The RNA‐seq analysis provides initial insights into the mechanisms contributing to the elevated abundance of specific metabolites in cord blood. However, further experimental investigation is required to fully elucidate the more comprehensive underlying mechanisms responsible for this distinct metabolic profile.

To investigate the antiaging efficacy of CF1, we first confirmed cellular uptake of its components via LC–MS quantification in human embryonic lung fibroblasts (MRC‐5 cells) posttreatment. Compared to controls, all five metabolites showed significantly increased intracellular levels, confirming successful uptake (Figure S4A–E). We then evaluated CF1's effects using two well‐established cellular senescence models in MRC‐5 cells. In the etoposide‐induced DNA damage–associated senescence mode, CF1 treatment significantly reduced the number of senescence‐associated β‐galactosidase (SA‐β‐Gal)‐positive cells (Figure 4B,C). At the molecular level, CF1 suppressed the upregulation of senescence‐related genes, including *p16*, *p21* (cell cycle inhibitors), and multiple SASP genes (*IL6*, *IL8*, *IL1α*, *IL1β*, *CXCL1*, *CXCL2*) (Figure 4D). In H_2_O_2_‐induced oxidative stress‐associated senescence mode, CF1 also reduced SA‐β‐Gal‐positive cells (Figure 4E–F). Transcriptomic analysis showed that CF1 significantly reduced the expression of cell cycle–related (*p16*, *p21*) and SASP genes, consistent with the DNA damage model (Figure 4G).

To further validate the antiaging potential of cord blood–derived candidate metabolites, we developed another formula, termed Cord Formula 2 (CF2), which comprises five short peptides (Lys Pro Asn, Lys Ala, Ile His, Lys Glu, and Phe Glu). They were chosen based on their high abundance in umbilical cord plasma relative to adult blood and age‐related decline (Figure S5A). Notably, they have no prior reports of antiaging activity. We evaluated their antiaging efficacy in stress‐induced senescence models using MRC‐5 cells. In the etoposide‐induced DNA damage‐associated senescence model, CF2 reduced the number of SA‐β‐Gal‐positive cells (Figure S5B,C) and suppressed the expression of cell cycle regulators and SASP genes (Figure S5D). Similar effects were observed in the H_2_O_2_‐induced oxidative stress‐associated senescence model, where CF2 decreased SA‐β‐Gal‐positive cells (Figure S5E,F) and downregulated *p16*, *p21* and SASP gene expression (Figure S5G).

Collectively, these findings demonstrate that two distinct cord blood‐derived formulations—CF1 and CF2—consistently mitigate stress‐induced senescence in human embryonic lung fibroblasts. Both formulas reduce senescence‐associated phenotypic markers (SA‐β‐Gal positivity) and suppress the transcriptional upregulation of core senescence drivers (*p16*, *p21*) and SASP genes across DNA damage and oxidative stress models. These results provide robust proof‐of‐concept that umbilical cord plasma harbors a repertoire of endogenous bioactive metabolites and peptides with intrinsic antisenescence activity.

### 2.6 Metabolites in Cord Blood Significantly Extending Lifespan and Healthspan in *C. elegans*

**FIGURE 5:**
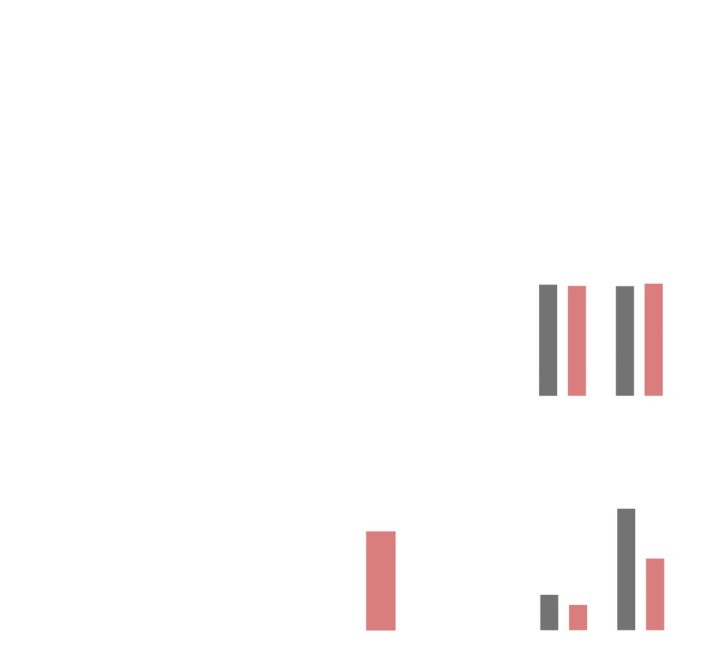
Significant lifespan and healthspan extension in *C. elegans* via CF1. (A) Lifespan of worms treated with CF1 or vehicle (H_2_O). *p* value represents comparison with vehicle calculated using long rank test. ***p < 0.001. (B) Representative intestinal autofluorescence and brightfield images (*n* = 16 images per group) of worms on days 8 and 12 of adulthood after being treated with CF1 or vehicle. The blue autofluorescence in the figure indicates the accumulation of lipofuscin. Scale bars, 300 μm. (C) The comparison of relative fluorescence intensity of intestinal autofluorescence in groups as indicated in B. (D) Body bending frequency on days 11 and 15 of adulthood in worms treated with CF1 or vehicle (*n* = 20). (E) Pharyngeal pumping frequency on days 8 and 12 of adulthood in worms treated with CF1 or vehicle (*n* = 20). (F) The body length of worms on days 8 and 12 was measured. (G) The total number of progenies from day1 to day 4 was summarized. (H) Survival for day 2 nematodes treated with CF1 or vehicle (H_2_O) was recorded after being exposed to 35°C heat shock for 7 h. (I) The nematodes on day 6 of adulthood treated with or without CF1 were harvested and measured for ROS levels by the fluorescent dye H2DCF‐DA (*n* = 500). For D‐F and I, *p* value was calculated by two‐way analysis of variance (ANOVA) followed by Bonferroni tests. For G‐H, *p* value was calculated by unpaired *t* test. *p < 0.05, **p < 0.01, ***p < 0.001; n.s., not significant; Values are mean ± SEM.

To further extrapolate the antisenescence effects of cord blood‐derived formulas from cellular models to a whole‐organism setting, we evaluated CF1and CF2 in the classic longevity research model *C. elegans*. For CF1, as shown in prior analyses, treatment at 100 μM significantly extended the lifespan of *C. elegans* compared to vehicle—treated controls (Figure 5A and Table S11). We then assessed CF2 in parallel. In the lifespan assay, CF2 treatment also significantly prolonged the lifespan of *C. elegans* (Figure S6A and Table S12). Lipofuscin, an age—associated autofluorescent pigment, was evaluated as a marker of aging. In both CF1 and CF2 treatments, lipofuscin accumulation was reduced (Figure 5B,C and Figure S6B,C). For CF2, while lipofuscin fluorescence intensity increased with age in vehicle—treated worms, CF2 effectively mitigated this accumulation from the middle (Day 12) to the late (Day 16) stages of adulthood (Figure S6B,C). Motor ability, which declines with age, was assessed by body bending rate and pharyngeal pumping rate. Both CF1 and CF2 treatments enhanced these parameters during aging. For CF2, as shown in Figure S6D,E, the body bending frequency and pharyngeal pumping rate were noticeably higher compared to the vehicle—treated group, consistent with the beneficial effect of CF1 (Figure 5D,E).

The safety of CF1 and CF2 was evaluated by examining body length and reproductive capacity. For both formulas, the body length of nematodes supplemented with CF1 or CF2 on days 8 and 12 remained consistent with that of the control group (Figure 5F and Figure S6F). In the fecundity assay, neither CF1 nor CF2 impaired the reproductive capacity of the worms (Figure 5G and Figure S6G). For CF2, similar to CF1, the number of progenies was not significantly different from the control, indicating that CF2, like CF1, enhances healthspan without affecting body length or fertility.

Adult worms were subjected to heat shock (35°C for 7 h) to assess stress resistance. Nematodes treated with CF1 or CF2 significantly outlived those in the control group (Figure 5H and Figure S6H). For CF2, this enhancement in stress resistance, particularly to heat shock, was comparable to that of CF1. Intracellular reactive oxygen species (ROS) levels, quantified using DCFH—DA, were significantly reduced by CF1treatment (Figure 5I). The reduction in ROS accumulation likely contributes to the observed benefits on longevity, fitness, and stress tolerance.

## 3 Discussion

Several studies have explored the metabolomics of umbilical cord blood, examining its connections with neonatal anthropometry and subsequent physiological and pathological indicators such as diabetes and autism (Guixeres‐Esteve et al. 2023; Kaupper et al. 2023; Oresic et al. 2013; Patel et al. 2018; Voerman et al. 2020; Xie et al. 2024; M. Zhang et al. 2022). However, no comparative metabolome analysis between umbilical cord blood and adult blood exists to date. Consequently, the unique metabolomic characteristics of umbilical cord blood remain largely unknown, leaving potential health‐beneficial, especially antiaging, metabolites undiscovered. In the present study, untargeted metabolomics analysis of human umbilical cord plasma (neonates) and plasma from young, middle‐aged, and older adults revealed a distinct metabolic landscape in cord blood. A total of 1092 metabolites were identified across all 210 plasma samples, including numerous compounds—such as lysine, threonine, isocitrate, xanthine, and kynurenic acid—that have been previously reported in adult plasma metabolomic studies (Cheng et al. 2015; Darst et al. 2019; Lawton et al. 2008; Montoliu et al. 2014; Sun et al. 2023; Yang and Kwon 2020). This overlap confirms the robustness of our analytical approach in capturing conserved metabolic features. Among the detected metabolites, 662 showed significantly differing abundance in cord plasma compared to adult plasma. Notably, 211 of these metabolites were enriched in cord blood and exhibited an age‐related decline, whose association with key aging‐related biological processes suggests potential antiaging properties.

Our global pathway investigation revealed extensive metabolic changes between cord and adult groups. Out of 59 mapped KEGG pathways, 43 showed significant differences (Figure 1E). Three of the top 10 differing pathways related to amino acid metabolism, including beta‐alanine metabolism, histidine metabolism and thiamine metabolism. These amino acid metabolic pathways have been reported to have health‐promoting benefits. Beta‐alanine metabolism is involved in the synthesis of carnosine, an antioxidant and neurotransmitter, improving exercise performance and reducing muscle fatigue (Cararo et al. 2015; Solana‐Manrique et al. 2022). Thiamine metabolism is crucial for ATP production, with deficiencies linked to neurological disorders like Alzheimer's disease (IuM et al. 1986). In addition to the aforementioned amino acid metabolic molecules, our study also revealed a significant increase in biotin metabolism and pantothenate and CoA biosynthesis in cord blood, which have been known for their beneficial roles in promoting wound healing and immune modulation (Gheita et al. 2020; Miallot et al. 2023).

To characterize prominent metabolic differences, we applied a 4‐fold abundance difference threshold (with adult groups analyzed collectively), identifying 56 metabolites with marked differences. Among these, 42 were cord blood‐enriched. Some of these differentially abundant metabolites hold known antiaging functions. For instance, carnosine (β‐alanyl‐L‐histidine) mitigates age‐associated biochemical changes like protein oxidation and advanced glycation end‐products (AGEs) formation (Cararo et al. 2015; Solana‐Manrique et al. 2022). Inosine, a purine nucleoside, has antioxidant, anti‐inflammatory effects and improves learning and memory capacity in aged rats (Elkouzi et al. 2019; Srinivasan et al. 2021). Although carnosine and inosines age‐related decline is known, our study is the first to identify their high abundance in cord blood—strengthening the link between cord‐enriched metabolites and known antiaging pathways.

We postulate antiaging metabolites are cord‐enriched and age‐declining. To identify such metabolites, a two‐tiered filtration approach was employed, resulting in the selection of 211 metabolites that met the criterion (see Table S7). To validate findings, we cross‐referenced 211 antiaging candidate metabolites against a number of independent age‐associated metabolomic resources (Collino et al. 2013; Ding et al. 2021; Menni et al. 2013; Sun et al. 2023; Yang et al. 2024; Yu et al. 2012; Zeng et al. 2024). This analysis identified 72 overlapping metabolites, with 52 showing an age‐related decline aligning with our untargeted screening. Metabolites like inosine, phosphocholine, L‐histidine, and L‐glutamine exhibited consistent age‐dependent reduction across multiple resources. The five core metabolites of CF1 (carnosine, inosine, L‐histidine, N‐acetylneuraminic acid, and taurocholic acid) also showed age‐related decline in these datasets. This convergence validates our analytical robustness and the biological relevance of cord blood‐enriched, age‐declining metabolites.

Aging is a universal process characterized by a decline in both structural homeostasis and functional integrity, leading to various health impairments including muscle mass and bone loss, cognitive decline, etc. (Cai et al. 2022; Fane and Weeraratna 2020; Hou et al. 2019). Several biological mechanisms are believed to contribute to the aging process and are known as aging hallmarks (Lopez‐Otin et al. 2023). Via literature review and functional annotation analysis, these 211 cord‐enriched metabolites were classified into well‐established aging‐related biological processes, including inflammation, oxidative stress, energy metabolism, proteostasis, and DNA damage response, further supporting their potential role in aging regulation. Additionally, literature review identified some of these metabolites with reported antiaging activity, such as uridine (Liu, Li, et al. 2022), niacinamide (Rajman et al. 2018) and taurine (Singh et al. 2023). We also identified metabolites like retinoic acid, which have functional annotations but have not yet been reported antiaging links. Notably, a number of metabolites, such as N1, N12‐Diacetylspermine, Ile His, and Asp Val Ser, have few functional annotations. Their potential involvement in antiaging processes remains entirely uncharacterized, offering fertile ground for discovering novel antiaging metabolites through future functional interrogations. Such investigations could reveal previously unrecognized molecules that modulate age regulation, thereby broadening our understanding of the metabolic pathways governing healthy aging.

Sex differences are a well‐established determinant of metabolic profiles in adult populations. For example, Burcu F. Darst et al. reported that 63.4% of plasma metabolites exhibited sex‐specific associations after multiple‐testing correction, with notable sex‐divergent trajectories in sphingolipids over aging (Darst et al. 2019). In this study, we implemented two strategies to systematically minimize sex‐related confounding factors. Firstly, participants were intentionally balanced by sex within each age group (Tables S1 and S2). Secondly, we explicitly excluded gonadal hormone‐related metabolites (e.g., estrogens, androgens, pregnenolones), which are directly regulated by sex steroids and exhibit pronounced dimorphism due to prenatal/maternal hormonal programming. While we did not perform subgroup analyses by sex, these strategies helped to ensure that observed differences (e.g., cord vs., adult plasma) reflect nonsex metabolic features. While these measures minimize sex‐related bias, residual dimorphisms may persist. Future large‐scale studies with sex‐stratified analyses will clarify these dynamics.

To validate the antiaging potential of these cord‐enriched metabolites, we formulated two blends (CF1 and CF2) and evaluated their effects in MRC5 fibroblasts and *C. elegans*. Our findings demonstrate that both formulations exert robust antiaging activity across these model systems. The consistent efficacy observed at both cellular and organismal levels provides compelling proof‐of‐concept, confirming that cord blood harbors bioactive components capable of mitigating aging through multitiered mechanisms. Endogenous metabolites offer inherent advantages for antiaging intervention development, including favorable bioavailability and safety profiles (Liu et al. 2025; Longo and Anderson 2022; Mahmoudi et al. 2019; Partridge et al. 2020). Notably, the cord blood‐enriched antiaging metabolites identified herein carry an additional layer of translational promise, given their natural presence in human neonates—a feature that may streamline future preclinical and clinical development.

Our current analysis of age‐specific metabolic profiles and candidate antiaging metabolites, while insightful, is constrained by the absence of systematic correlations with clinical and functional metrics. To build on the present work, future investigations could benefit from strategic expansion of cohort sizes—with particular emphasis on longevity populations—to capture the full spectrum of aging trajectories, including those of exceptional survivors with distinct metabolic signatures of healthy aging. Equally critical would be the integration of comprehensive health indicators beyond chronological age, such as detailed comorbidity assessments (e.g., hypertension, diabetes prevalence), objective physical function measurements (e.g., grip strength, gait speed), and cognitive evaluations (e.g., memory, executive function). By anchoring metabolite profiles to these multidimensional outcomes, such follow‐up studies could rigorously validate the association between identified metabolites and healthy aging trajectories, transcending the limitations of our current correlative analyses and clarifying their specific roles in sustaining physiological resilience. Ultimately, such efforts may distinguish whether these metabolites are mere markers of aging or active modulators of healthy aging processes—insights that could guide the identification of specific molecules with the potential to extend healthspan and lifespan. To fully realize this potential, future work should focus on elucidating their mechanistic roles in aging pathways and exploring strategies to enhance their bioavailability in vivo, such as targeted dietary interventions, lifestyle modifications, or precision supplementation. These endeavors could pave the way for novel antiaging therapies, laying the groundwork for interventions that enhance longevity while preserving functional vitality.

## 4 Methods

### 4.1 Plasma Sample Preparation

Umbilical cord blood was collected immediately postdelivery via the umbilical vein, and adult blood samples were collected after an overnight fast (≥ 8 h) between 8:00 AM and 10:00 AM to minimize diurnal variation. For plasma collection, blood was centrifuged at 3000 *g* at 4°C for 20 min. The plasma samples were aliquoted and stored at −80°C until execution of metabolic analyses.

### 4.2 UPLC‐ESI‐MS/MS Analysis

Plasma metabolites were extracted using organic solvents. Plasma (100 μL) was treated with four volumes (400 μL) of an acetonitrile: methanol (1:1, v/v) solvent mixture, mixed and incubated at −20°C for 1 h to allow for protein precipitation. The supernatant was collected after centrifugation at 12000 rpm for 15 min at 4°C and evaporated under nitrogen to dryness. Metabolites were analyzed using the TripleTOF 6600 hybrid Q‐TOF mass spectrometer (SCIEX, Foster City, CA, USA) equipped with a TurboVTM electrospray interface. The mass spectrometer was coupled to a Shimadzu Prominence UPLC system LC‐30 ad (Shimadzu, Kyoto, Japan). For the analysis, two columns were utilized: the ACQUITY UPLC HSS T3 column (2.1 mm × 100 mm, 1.8 μm) and the ACQUITY BEH amide column (2.1 mm × 100 mm, 1.7 μm), both of which were obtained from Waters (Waters, Ireland). Metabolic extracts were analyzed by the ultrahigh‐performance liquid chromatography system coupled to hybrid Q‐TOF mass spectrometry (UPLC‐QTOF‐MS) in both positive and negative ionization modes. To increase the metabolome coverage, the metabolite separation was performed on an HSS T3 column and a BEH amide column, respectively. The TripleTOF 6600 mass spectrometer was operated in full MS‐scan mode/data‐dependent analysis (DDA) mode in both positive and negative modes. The precursor ion and fragment ion mass‐to‐charge ratio (*m/z*) scan range was set as 50–1000 Da for both. The accumulation time for MS1 was 150 ms, while for MS2 it was 50 ms. The collision energy (CE) voltage was sequentially set at 15 *eV*, 30 *eV*, and 45 *eV* for testing parameters. Gas1 and gas2 were maintained at 50 *Pa*, while the curtain gas was set at 35 Pa. The desolvation gas temperature and source temperature were both set to 500°C. The ion spray voltage floating (ISVF) was 5.5 *kV* for ESI+ mode and −4.5 *kV* for ESI‐ mode. The declustering potential (DP) was 80 *V* for ESI+ mode and −80 *V* for ESI‐ mode. Each sample was analyzed in the four modes, with three technical replicates (three injections) performed for each. For all samples in each batch, the median intensity of each metabolite across samples is calculated. This median value is then used to divide the original metabolite intensities in all samples so that the median value of each metabolite across all samples within the batch will be equal to 1 after scaling. To monitor the consistency of retention time and signal intensity, a quality control (QC) sample was created by pooling plasma samples from 10 individuals in each group. This QC sample was injected between every 10 sample injections.

### 4.3 Metabolomics Data Processing and Analysis

The Progenesis QI software (version 3.0.3, Waters, Milford, MA, USA) was utilized for data processing. To minimize the inclusion of false positives originating from early‐eluting compounds or instrumental artifacts, features with retention times less than 30s were filtered out. In order to ensure the reliability of our analysis, we required that a feature be present in at least 70% of the samples within at least one of the four age groups, thus avoiding the inclusion of features with low abundance across all groups.

For metabolite identification, we employed two widely used metabolite databases: HMDB and METLIN. The former was accessed directly through the HMDB website (https://www.hmdb.ca/) and the latter was acquired from Waters Company (Waters, Milford, MA, USA). The QI identification score, which is based on accurate mass, fragment pattern, and isotope similarity, could accumulate up to 60 points, with each aspect contributing a maximum of 20. Metabolites matched to the HMDB database were putatively identified if they had QI scores of 47 or higher and a fragmentation score of at least 50. For those matched to the METLIN database, putative identification required a score of 50 or more and a fragmentation score of at least 40. To ensure the reliability of the identifications, the metabolites were also manually verified by comparing the measured tandem spectra with the experimental spectra from METLIN or the predictable spectra from HMDB. The mass tolerance for both precursor and fragment ions was set at 12 ppm. The database search was performed prior to the pattern recognition analysis.

We implemented PCA to depict the distribution of the sample metabolome in the low dimensional embedding, based on the 59,690 identified features. The aging trajectory was superimposed on the PCA plot to facilitate the analysis. The samples were separated by age group in the PCA space which is defined by two components that explained the largest variations (PC1 and PC2, Figure 1B).

### 4.4 Pathway Analysis

We employed MetaboAnalystR to conduct the Metabolite Set Enrichment Analysis (MSEA) on the identified metabolites. To quantify pathway activity, we computed the average intensities of all identified metabolites for each pathway and represented them on a heatmap. The statistical significance of the changes in the activity of a pathway across age groups was assessed by global testing, the default method utilized by MetaboAnalystR. The topological impacts on the pathway were quantified using MetaboAnalystR.

### 4.5 Network Analysis

A regularized partial correlation network was generated to capture the remaining association between two nodes after controlling all other information (indirect correlations) within the network. In this network, each node symbolizes a compound, with each edge representing the strength of the partial correlation between two nodes, conditioned on all other variables in the datasets. The weights of the edges correspond to the partial correlation coefficients (as illustrated in Figure 2A). To confine spurious correlations within the network, we employed the Lasso (Least Absolute Shrinkage and Selection Operator) method, which shrinks smaller association coefficient values to zero. The implementation of the Lasso‐based regularized partial correlation was achieved using the qgraph package in R. The importance of metabolites within the network was determined by three metrics: strength (the cumulative sum of absolute edge weights connected to each metabolite), closeness (the inverse of the total distances from one metabolite to all others), and betweenness (the frequency of a metabolite appearing in the shortest paths between other metabolites).

### 4.6 Gene Expression Analysis of Metabolic Associated Enzymes

To investigate transcriptional levels of metabolite synthesis and hydrolysis enzymes across cord blood, young adult, and elderly cohorts, we analyzed two publicly available peripheral blood mononuclear cell (PBMC) RNA sequencing datasets: (1) cord blood PBMC RNA‐seq (GEO accession: GSE136849) and (2) adult PBMC RNA‐seq (GEO accession: GSE153100). These datasets include demographic and clinical characteristics of participants, as detailed in the original publications (Chen et al. 2020; Wahl et al. 2021). In total, our analysis incorporated 6 cord blood samples from healthy neonates, 5 young adults (18–22 years, 2 males/3 females), and 5 older adults (62–74 years, 2 males/3 females).

### 4.7 Reagents

5‐Fluoro‐20‐deoxyuridine (FUDR), 20,70‐dichlorofluorescin diacetate (DCFH‐DA), Inosine (I4125) and Carnosine (C9625) were purchased from Sigma‐Aldrich Co. (St. Louis, MO, USA). Taurocholic acid (HY‐B1788) was purchased from MedChemExpress LLC (New Jersey, USA). L‐Histidine (A070079) and N‐acetylneuraminic acid (A010508) were purchased from Beijing Energy Engineering Technologies Co. Ltd. (Beijing, China). Lys‐Pro‐Asn, Lys‐Ala, Ile‐His, Lys‐Glu and Phe‐Glu were synthesized by Synpeptide Co. Ltd. (Nanjing, China).

### 4.8 Cell Culture and Senescence Induction

MRC‐5 cells were cultured in MEM medium supplemented with 10% FBS, penicillin (100 U/mL), and streptomycin (100 mg/mL). For H₂O₂‐induced senescence, cells were pretreated with CF1, CF2 or left untreated, followed by exposure to 200 μM H_2_O_2_ for 1 h. After washing, cells were continuously cultured in complete medium for 3 days. For etoposide‐induced senescence, cells were pretreated with CF1, CF2 or left untreated. Following pretreatment, cells were exposed to 10 μM etoposide for 24 h, washed, and subsequently cultured in complete medium for an additional 5 days before collection.

### 4.9 SA‐β‐Gal Staining

SA‐β‐gal activity was detected using the Senescence β‐Galactosidase staining kit (Cell Signaling Technology, #9860) according to the manufacturer's instructions with minor modifications. Briefly, MRC‐5 cells were washed twice with PBS and fixed with fixative solution for 15 min at room temperature. Following fixation, cells were rewashed with PBS and incubated in freshly prepared staining solution [1 mg/mL X‐gal, 5 mM potassium ferrocyanide, 150 mM NaCl, 2 mM MgCl₂, and 2% (v/v) N,N‐dimethylformamide (DMF), pH 6.0] at 37°C in a CO₂‐free incubator for 12–16 h. Stained cells were imaged using a Thermo EVOS 5000 fluorescence imaging system, and the number of SA‐β‐gal‐positive cells was quantified using ImageJ software.

### 4.10 RNA Extraction and qPCR

Total RNA was extracted from harvested cells using TRIzol reagent (Sigma‐Aldrich, T9424) according to the manufacturer's instructions. Reverse transcription of 0.5 μg total RNA was performed using PrimeScript RT Master Mix (Takara, DRR036A). qPCR was conducted using the FastStart Universal SYBR Green Master (Rox) (Roche, 04913914001) to determine relative mRNA levels. The gene expression was then normalized to that of GAPDH. Primer sequences used for qPCR analysis are listed in Table S13.

### 4.11 Evaluation of Nematode Lifespan and Healthspan

The wild‐type (N2) *C. elegans* strain and *Escherichia coli* OP50 ( *E. coli* OP50) were procured from the Hong Zhang laboratory at the University of Chinese Academy of Sciences in Beijing, China. Lifespan and healthspan evaluation were conducted as previously described (Liu, Zheng, et al. 2022). In brief, stock solutions of CF1 and CF2 were individually mixed with *E. coli* OP50, such that each of the five constituent metabolites in both formulations reached a final concentration of 100 μM. Approximately 100 synchronized nematodes in the L4 stage were used for the experiment. In total, 5 μM of FUDR was added into the NGM plates to inhibit progeny production for a period of 10 days, after which the worms were relocated to ordinary NGM plates devoid of FUDR. Nematodes were counted daily until all had perished. On the 8th, 12th or 16th days of adulthood, the nematodes were anesthetized using 10 mM levamisole hydrochloride and subsequently secured on a 2% agarose plate. The intestinal fluorescence was observed using a Thermo EVSO 5000 fluorescence microscope, and lipofuscin levels were quantified using ImageJ software. Each group analyzed comprised more than 15 worms. To determine body bending frequencies, nematodes on day 11 and day 15 of adulthood were relocated to new, *E. coli* ‐free NGM plates and given a 1‐min acclimation period. Body bending frequencies were subsequently recorded over a 30‐s span. Each group consisted of approximately 20 nematodes. The frequency of pharyngeal pumping was evaluated on the plates on day 8 and day 12 of adulthood. The number of pumps was recorded over a 30‐s duration, with each group consisting of approximately 20 nematodes. To determine body length, nematodes on day 8 and day 12 of adulthood were anesthetized using 10 mM levamisole hydrochloride and affixed on a 2% agarose plate. The nematodes were then visualized using a bright‐field microscope at ×10 magnification. The body length was measured using ImageJ software, with each group comprising more than 15 worms for examination. For the fecundity assay, nematodes at the L4 larval stage were relocated to fresh NGM plates every 24 h postegg‐laying. Each plate contained only one nematode, with more than 15 worms examined. This process was consistently repeated during the initial 4 days of the lifespan. After a 3‐day incubation period at 20°C, the daily progeny count was recorded, and subsequently, the total progeny count was computed.

The resistance of nematodes to heat stress was determined as previously described (Liu, Zheng, et al. 2022). In brief, 6‐day‐old nematodes, both those treated with 100 μM CF and untreated controls, were exposed to 35°C for 7 h. Following this, the survival rate of these nematodes (*n* = 100) was recorded on an hourly basis, with this observation persisting until all the nematodes had expired. To determine the ROS in *C. elegans*, approximately 500 nematodes were gathered and ultrasonicated for 20 min until the nematodes were no longer visible. The supernatant was collected and combined with 50 μL of DCFH‐DA. Fluorescence was then detected using a fluorescence microplate reader (Molecular Devices, San Jose, CA, USA) with an excitation wavelength of 485 nm and an emission wavelength of 538 nm. All experiments involving *C. elegans* were conducted at least three times.

### 4.12 Statistical Analysis

PCA was employed for dimensionality reduction of all samples based on LC–MS features. A two‐tailed *t*‐test was utilized to identify metabolites with differential intensities between cord and adult groups. The statistical significance and topological impacts of pathway activity changes across age groups were assessed using global testing implemented with the MetaboAnalystR package. The association between metabolite intensities in all samples and the corresponding age was quantified using Pearson's correlation coefficient.

In the *C. elegan*s associated experiments, data are presented as the mean ± SEM. *p* value for survival curves were calculated using the log‐rank test. For MRC‐5 cell experiments, data are presented as the mean ± SD. Statistical analyses to assess significant differences (defined as *p* < 0.05) were performed using GraphPad Prism software, employing either two‐way analysis of variance (ANOVA) followed by Bonferroni post hoc tests, or unpaired *t*‐test, as appropriate.

## Author Contributions

Qing Xia, WeiNa Zhang and Na Wang supervised the research. JiaYu Liu, YanYan Shen and Zhi Jin carried out the experiments. Shuai Jiang carried out most of the bioinformatic analysis. RuiBo Wang, Zhi Jin, JinLiang Li, YanHong Liu, Qi Qi, Yue Guo, BoYang Xie, YunYing Wang, JunCheng Li, AiPing Cao, Yao Wang and ChunYan Yan collected and processed clinical blood samples and medical information. YanQing Cao, YingJie Zhu, Jing Peng, FangTing Dong and Kun He performed mass spectrometry analysis. Xin Pan, XinHua He, Tao Zhou, AiLing Li, Na Wang, WeiNa Zhang and Qing Xia contributed to interpreting the results and providing intellectual support. Shuai Jiang, JiaYu Liu, WeiNa Zhang and Qing Xia wrote the manuscript.

## Conflicts of Interest

The authors declare no conflicts of interest.

## Supporting information


**Appendix S1:** acel70295‐sup‐0001‐AppendixS1.pptx.

**Appendix S2:** acel70295‐sup‐0002‐AppendixS2.docx.

**Table S3:** acel70295‐sup‐0003‐TableS3.xlsx.

**Table S4:** acel70295‐sup‐0004‐TableS4.xlsx.

**Table S5:** acel70295‐sup‐0005‐TableS5.xlsx.

**Table S6:** acel70295‐sup‐0006‐TableS6.xlsx.

**Table S7:** acel70295‐sup‐0007‐TableS7.xlsx.

**Table S8:** acel70295‐sup‐0008‐TableS8.xlsx.

**Table S9:** acel70295‐sup‐0009‐TableS9.xlsx.

**Table S10:** acel70295‐sup‐0010‐TableS10.xlsx.

## Data Availability

The data that supports the findings of this study are available in the Supporting Information of this article.
