# Supplementary material for: Human Umbilical Cord Plasma Metabolomics Uncover Potential Metabolites for Combating Aging
Source: Aging Cell. 2025 Nov 26;25(1):e70295. doi: 10.1111/acel.70295 (PMC12740092; doi:10.1111/acel.70295)
Supplement: Supplementary file 1 — Appendix S1: acel70295‐sup‐0001‐AppendixS1.pptx. [file ACEL-25-e70295-s002.pptx]

## Slide 1
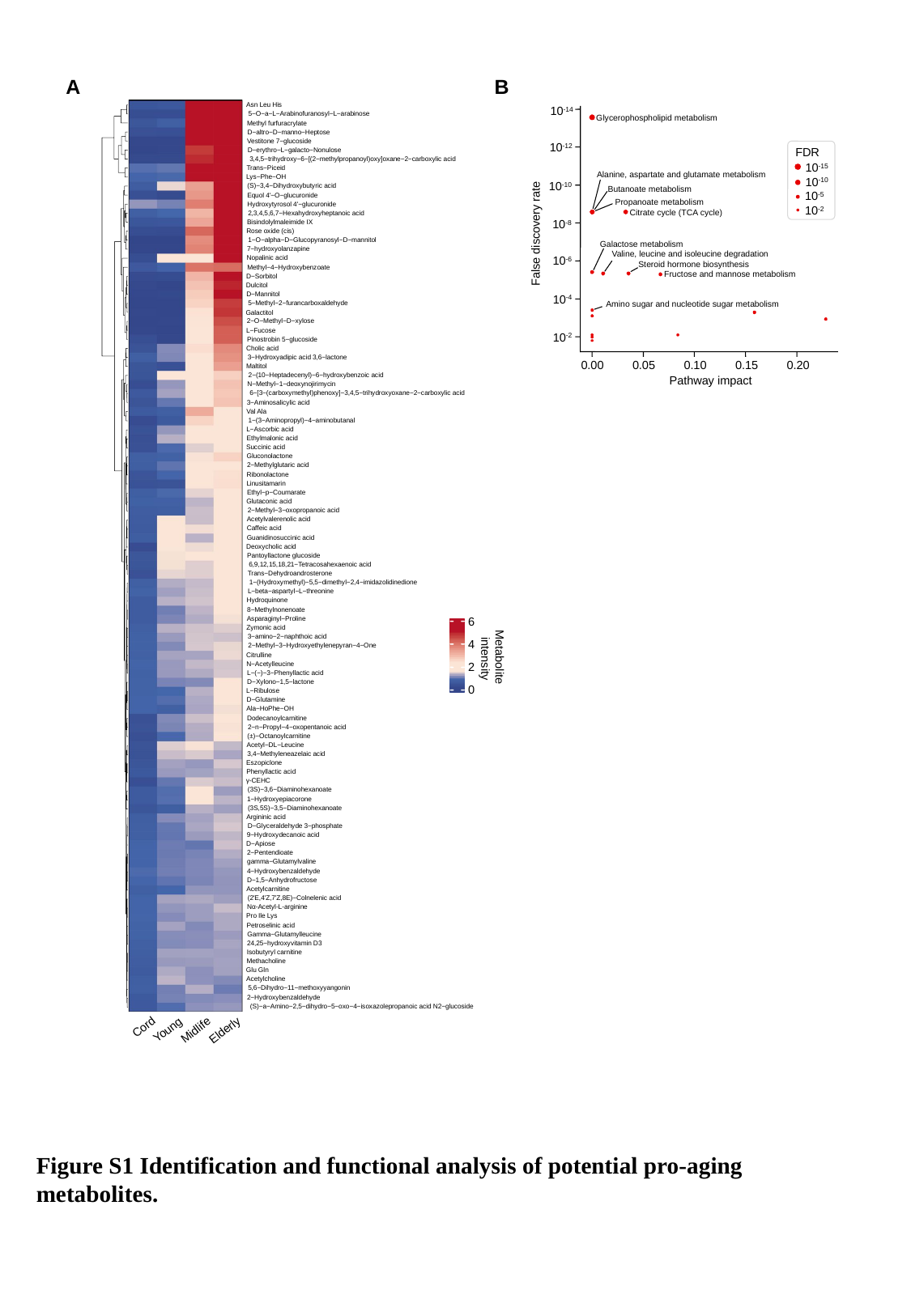

A
B
Asn Leu His
5−O−a−L−Arabinofuranosyl−L−arabinose
Methyl furfuracrylate
D−altro−D−manno−Heptose
Vestitone 7−glucoside
D−erythro−L−galacto−Nonulose
3,4,5−trihydroxy−6−[(2−methylpropanoyl)oxy]oxane−2−carboxylic acid
Trans−Piceid
Lys−Phe−OH
(S)−3,4−Dihydroxybutyric acid
Equol 4'−O−glucuronide
Hydroxytyrosol 4'−glucuronide
2,3,4,5,6,7−Hexahydroxyheptanoic acid
Bisindolylmaleimide IX
Rose oxide (cis)
1−O−alpha−D−Glucopyranosyl−D−mannitol
7−hydroxyolanzapine
Nopalinic acid
Methyl−4−Hydroxybenzoate
D−Sorbitol
Dulcitol
D−Mannitol
5−Methyl−2−furancarboxaldehyde
Galactitol
2−O−Methyl−D−xylose
L−Fucose
Pinostrobin 5−glucoside
Cholic acid
3−Hydroxyadipic acid 3,6−lactone
Maltitol
2−(10−Heptadecenyl)−6−hydroxybenzoic acid
N−Methyl−1−deoxynojirimycin
6−[3−(carboxymethyl)phenoxy]−3,4,5−trihydroxyoxane−2−carboxylic acid
3−Aminosalicylic acid
Val Ala
1−(3−Aminopropyl)−4−aminobutanal
L−Ascorbic acid
Ethylmalonic acid
Succinic acid
Gluconolactone
2−Methylglutaric acid
Ribonolactone
Linusitamarin
Ethyl−p−Coumarate
Glutaconic acid
2−Methyl−3−oxopropanoic acid
Acetylvalerenolic acid
Caffeic acid
Guanidinosuccinic acid
Deoxycholic acid
Pantoyllactone glucoside
6,9,12,15,18,21−Tetracosahexaenoic acid
Trans−Dehydroandrosterone
1−(Hydroxymethyl)−5,5−dimethyl−2,4−imidazolidinedione
L−beta−aspartyl−L−threonine
Hydroquinone
8−Methylnonenoate
Asparaginyl−Proline
Zymonic acid
3−amino−2−naphthoic acid
2−Methyl−3−Hydroxyethylenepyran−4−One
Citrulline
N−Acetylleucine
L−(−)−3−Phenyllactic acid
D−Xylono−1,5−lactone
L−Ribulose
D−Glutamine
Ala−HoPhe−OH
Dodecanoylcarnitine
2−n−Propyl−4−oxopentanoic acid
(±)−Octanoylcarnitine
Acetyl−DL−Leucine
3,4−Methyleneazelaic acid
Eszopiclone
Phenyllactic acid
γ-CEHC
(3S)−3,6−Diaminohexanoate
1−Hydroxyepiacorone
(3S,5S)−3,5−Diaminohexanoate
Argininic acid
D−Glyceraldehyde 3−phosphate
9−Hydroxydecanoic acid
D−Apiose
2−Pentendioate
gamma−Glutamylvaline
4−Hydroxybenzaldehyde
D−1,5−Anhydrofructose
Acetylcarnitine
(2'E,4'Z,7'Z,8E)−Colnelenic acid
Nα-Acetyl-L-arginine
Pro Ile Lys
Petroselinic acid
Gamma−Glutamylleucine
24,25−hydroxyvitamin D3
Isobutyryl carnitine
Methacholine
Glu Gln
Acetylcholine
5,6−Dihydro−11−methoxyyangonin
2−Hydroxybenzaldehyde
(S)−a−Amino−2,5−dihydro−5−oxo−4−isoxazolepropanoic acid N2−glucoside
10-14
Glycerophospholipid metabolism
10-12
FDR
10-15
10-10
10-5
10-2
Alanine, aspartate and glutamate metabolism
10-10
Butanoate metabolism
Propanoate metabolism
Citrate cycle (TCA cycle)
10-8
False discovery rate
Galactose metabolism
Valine, leucine and isoleucine degradation
10-6
Steroid hormone biosynthesis
Fructose and mannose metabolism
10-4
Amino sugar and nucleotide sugar metabolism
10-2
0.00
0.05
0.10
0.15
0.20
Pathway impact
6
4
Metabolite
intensity
2
0
Cord
Young
Midlife
Elderly
Figure S1 Identification and functional analysis of potential pro-aging metabolites.

## Slide 2
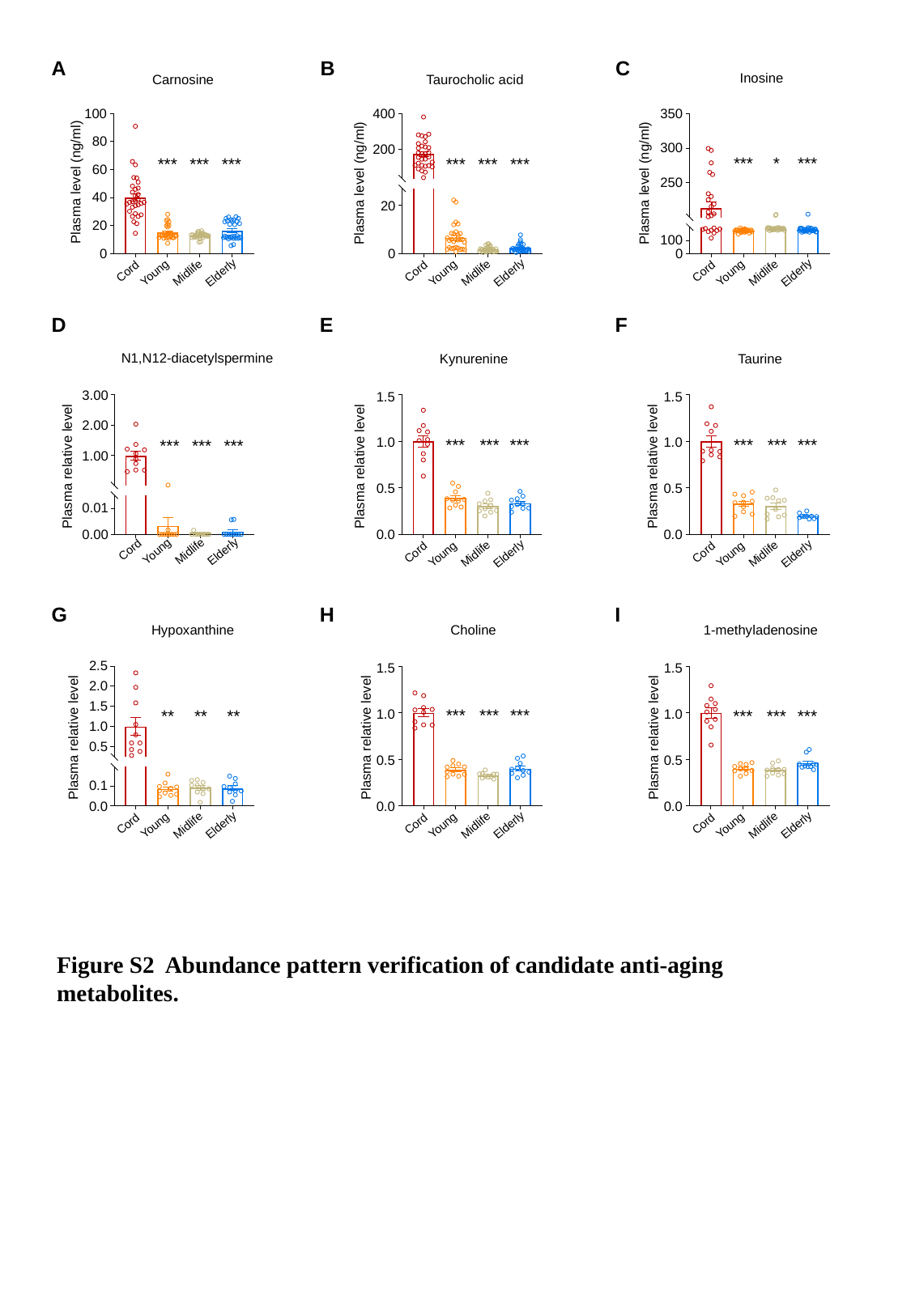

A
B
C
Inosine
350
300
***
*
***
250
Plasma level (ng/ml)
100
0
Cord
Midlife
Young
Elderly
Carnosine
100
80
***
***
***
60
Plasma level (ng/ml)
40
20
0
Cord
Midlife
Young
Elderly
Taurocholic acid
400
200
***
***
***
Plasma level (ng/ml)
20
0
Cord
Midlife
Young
Elderly
D
E
F
N1,N12-diacetylspermine
3.00
2.00
***
***
***
1.00
Plasma relative level
0.01
0.00
Cord
Midlife
Young
Elderly
Kynurenine
1.5
***
***
***
1.0
Plasma relative level
0.5
0.0
Cord
Midlife
Young
Elderly
Taurine
1.5
***
***
***
1.0
Plasma relative level
0.5
0.0
Cord
Midlife
Young
Elderly
G
H
I
Hypoxanthine
2.5
2.0
1.5
**
**
**
1.0
Plasma relative level
0.5
0.1
0.0
Cord
Midlife
Young
Elderly
Choline
1.5
***
***
***
1.0
Plasma relative level
0.5
0.0
Cord
Midlife
Young
Elderly
1-methyladenosine
1.5
***
***
***
1.0
Plasma relative level
0.5
0.0
Cord
Midlife
Young
Elderly
Figure S2 Abundance pattern verification of candidate anti-aging metabolites.

## Slide 3
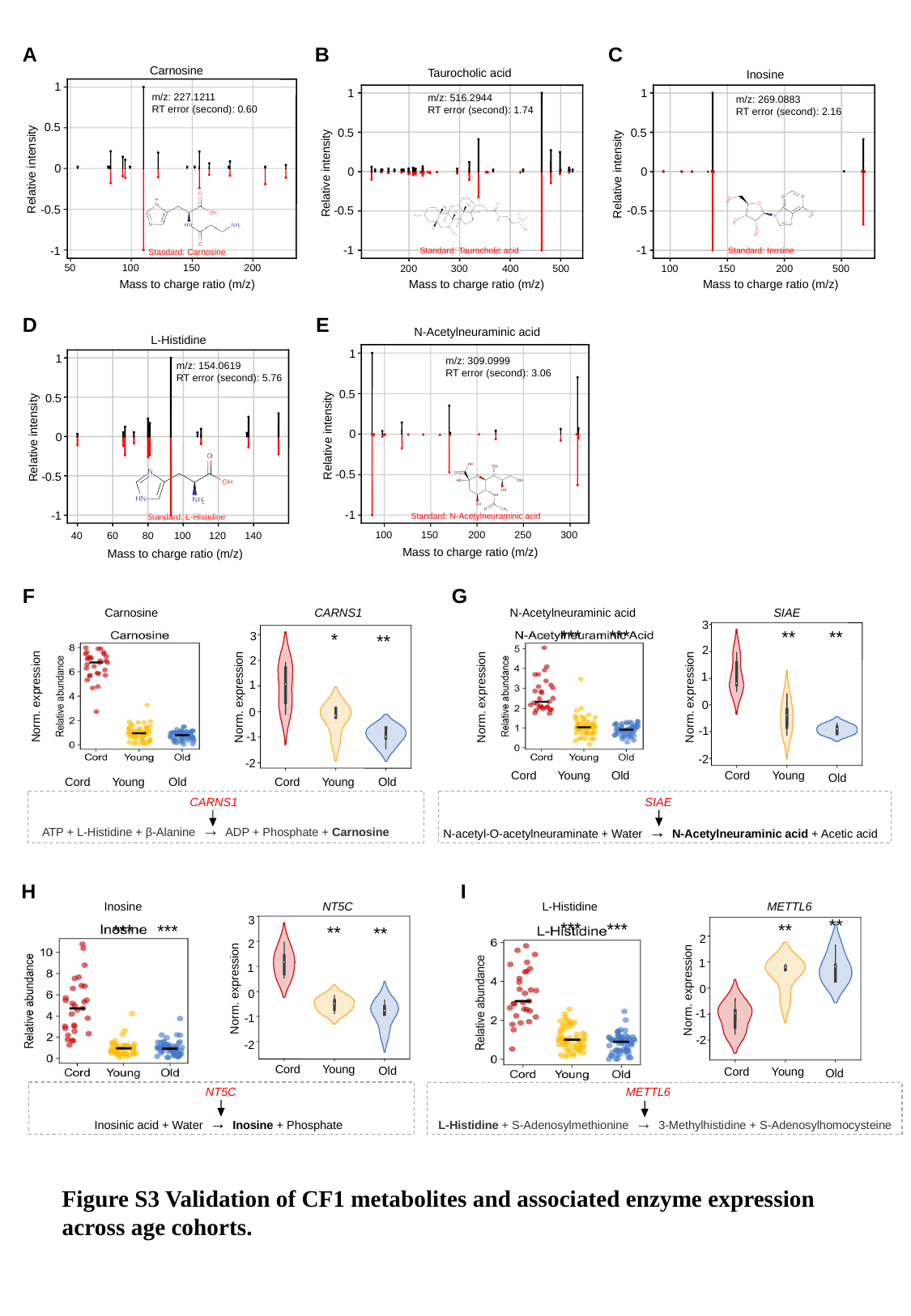

A
B
C
Carnosine
1
m/z: 227.1211
RT error (second): 0.60
0.5
0
-0.5
-1
Standard: Carnosine
50
100
150
200
Relative intensity
Mass to charge ratio (m/z)
Taurocholic acid
1
m/z: 516.2944
RT error (second): 1.74
0.5
0
-0.5
-1
Standard: Taurocholic acid
200
300
400
500
Relative intensity
Mass to charge ratio (m/z)
Inosine
1
m/z: 269.0883
RT error (second): 2.16
0.5
0
-0.5
-1
Standard: Inosine
100
150
200
500
Relative intensity
Mass to charge ratio (m/z)
D
E
N-Acetylneuraminic acid
1
m/z: 309.0999
RT error (second): 3.06
0.5
0
-0.5
-1
Standard: N-Acetylneuraminic acid
100
150
200
250
300
Relative intensity
Mass to charge ratio (m/z)
L-Histidine
1
m/z: 154.0619
RT error (second): 5.76
0.5
0
-0.5
-1
40
60
80
100
120
140
Relative intensity
Mass to charge ratio (m/z)
Standard: L-Histidine
F
G
Carnosine
Norm. expression
Cord
Young
Old
CARNS1
*
3
**
2
1
Norm. expression
0
-1
-2
Cord
Young
Old
N-Acetylneuraminic acid
***
***
Norm. expression
Cord
Young
Old
SIAE
3
2
1
0
-1
-2
**
**
Norm. expression
Cord
Young
Old
***
***
SIAE
CARNS1
ATP + L-Histidine + β-Alanine → ADP + Phosphate + Carnosine
N-acetyl-O-acetylneuraminate + Water → N-Acetylneuraminic acid + Acetic acid
H
I
Inosine
***
***
NT5C
3
2
1
0
-1
-2
**
**
Norm. expression
Cord
Young
Old
L-Histidine
***
***
METTL6
**
**
2
1
0
-1
-2
Norm. expression
Cord
Young
Old
NT5C
METTL6
Inosinic acid + Water → Inosine + Phosphate
L-Histidine + S-Adenosylmethionine → 3-Methylhistidine + S-Adenosylhomocysteine
Figure S3 Validation of CF1 metabolites and associated enzyme expression across age cohorts.

## Slide 4
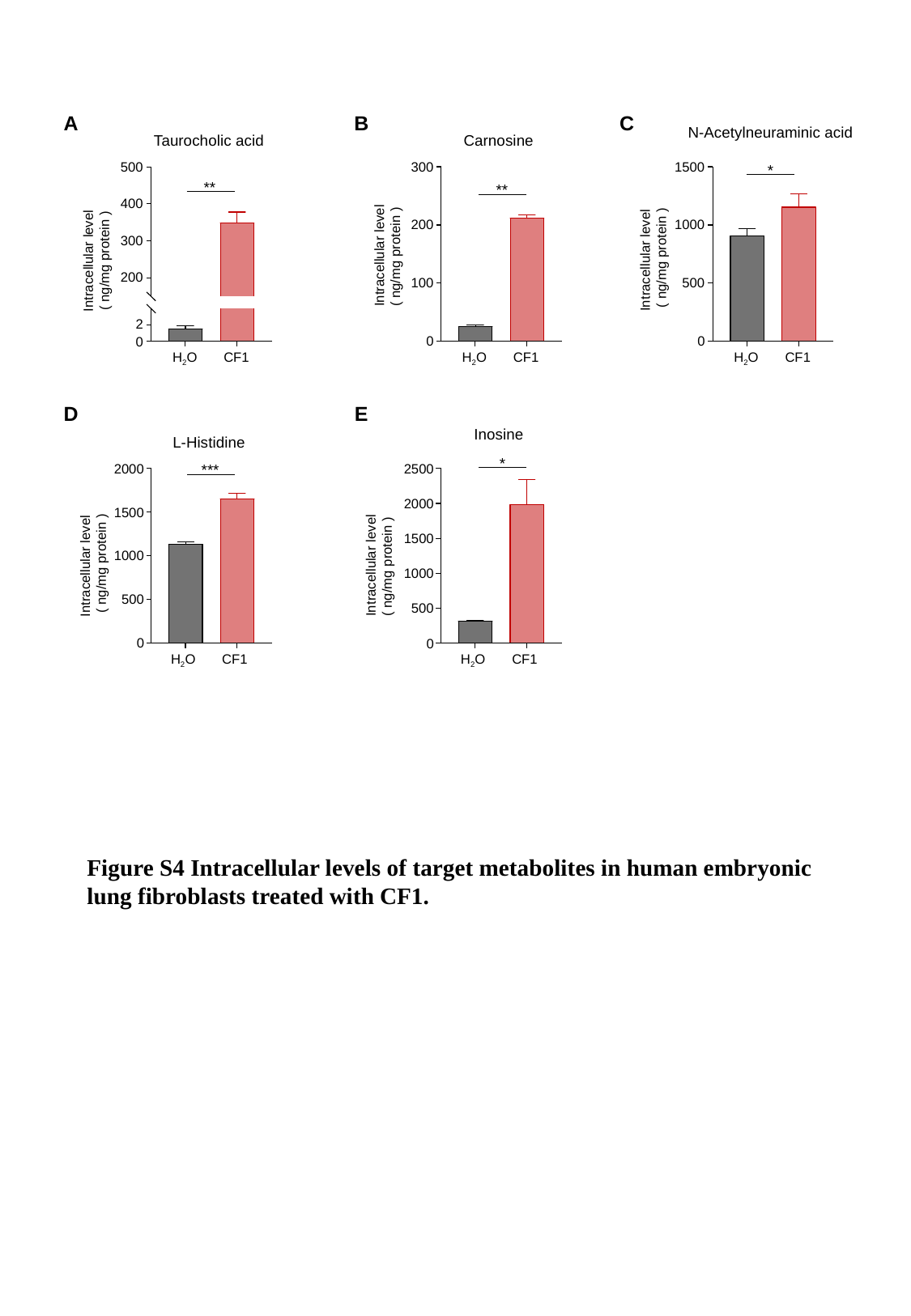

A
B
C
N-Acetylneuraminic acid
*
1500
1000
Intracellular level
 ( ng/mg protein )
500
0
H2O
CF1
Taurocholic acid
500
**
400
300
Intracellular level
( ng/mg protein )
200
2
0
H2O
CF1
Carnosine
300
**
200
Intracellular level
( ng/mg protein )
100
0
H2O
CF1
D
E
Inosine
*
2500
2000
1500
Intracellular level
( ng/mg protein )
1000
500
0
H2O
CF1
L-Histidine
***
2000
1500
Intracellular level
 ( ng/mg protein )
1000
500
0
H2O
CF1
Figure S4 Intracellular levels of target metabolites in human embryonic lung fibroblasts treated with CF1.

## Slide 5
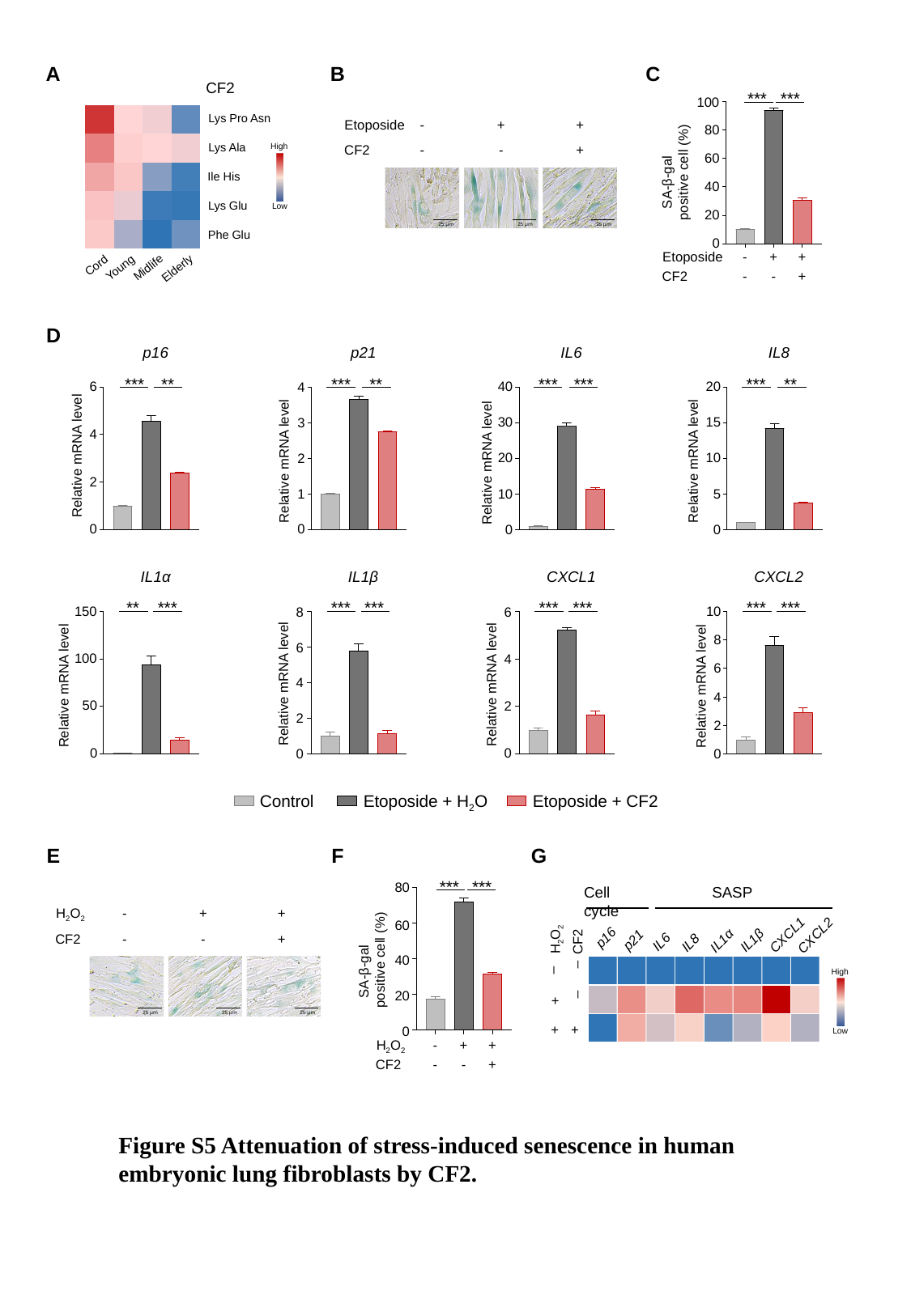

A
B
C
CF2
***
***
100
80
60
SA-β-gal
positive cell (%)
40
20
0
Etoposide
-
+
+
CF2
-
-
+
| | | | |
| --- | --- | --- | --- |
| | | | |
| | | | |
| | | | |
| | | | |
Lys Pro Asn
Etoposide
-
+
+
CF2
-
-
+
25 μm
25 μm
25 μm
High
Low
Lys Ala
Ile His
Lys Glu
Phe Glu
Cord
Young
Midlife
Elderly
D
p16
***
**
6
4
Relative mRNA level
2
0
p21
***
**
4
3
2
Relative mRNA level
1
0
IL6
***
***
40
30
20
Relative mRNA level
10
0
IL8
***
**
20
15
10
Relative mRNA level
5
0
IL1α
**
***
150
100
Relative mRNA level
50
0
IL1β
***
***
8
6
4
Relative mRNA level
2
0
CXCL1
***
***
6
4
Relative mRNA level
2
0
CXCL2
***
***
10
8
6
Relative mRNA level
4
2
0
Control
Etoposide + H2O
Etoposide + CF2
E
F
G
***
***
80
60
SA-β-gal
positive cell (%)
40
20
0
H2O2
-
+
+
CF2
-
-
+
Cell cycle
SASP
p16
IL1β
CXCL1
CXCL2
p21
IL6
H2O2
IL8
IL1α
CF2
_
_
High
_
+
+
+
Low
H2O2
-
+
+
CF2
-
-
+
25 μm
25 μm
25 μm
Figure S5 Attenuation of stress-induced senescence in human embryonic lung fibroblasts by CF2.

## Slide 6
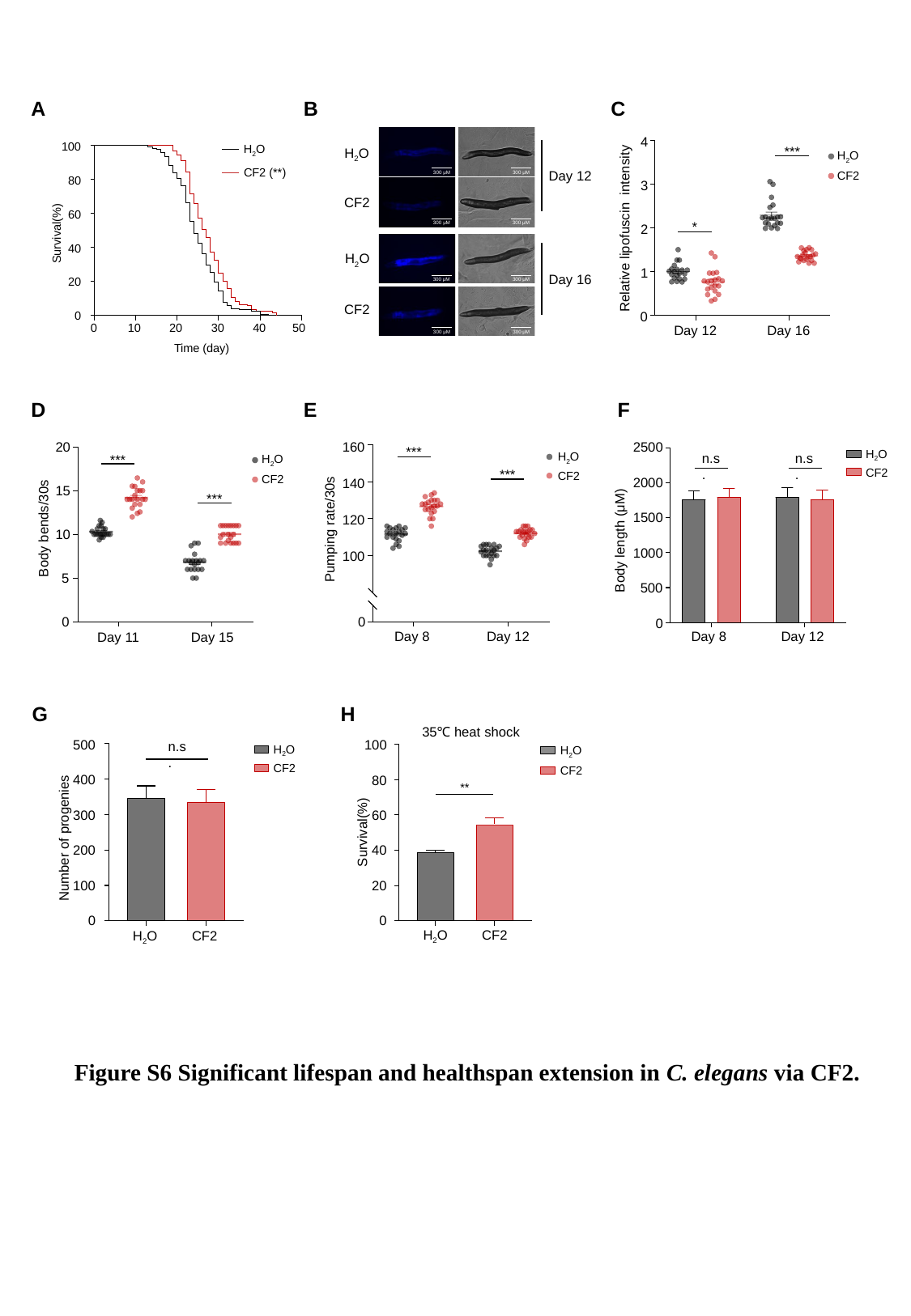

A
B
C
100
H2O
CF2 (**)
80
60
40
Survival(%)
20
0
0
10
20
30
40
50
Time (day)
4
***
H2O
CF2
3
*
Relative lipofuscin intensity
2
1
0
Day 12
Day 16
300 μM
300 μM
H2O
Day 12
300 μM
300 μM
CF2
300 μM
300 μM
H2O
Day 16
300 μM
300 μM
CF2
D
E
F
160
***
H2O
CF2
***
140
120
Pumping rate/30s
100
0
Day 8
Day 12
20
***
H2O
CF2
15
***
Body bends/30s
10
5
0
Day 11
Day 15
2500
H2O
CF2
n.s.
n.s.
2000
1500
Body length (μM)
1000
500
0
Day 8
Day 12
G
H
500
n.s.
H2O
400
CF2
300
200
Number of progenies
100
0
H2O
CF2
100
35℃ heat shock
H2O
80
CF2
**
60
40
Survival(%)
20
0
H2O
CF2
Figure S6 Significant lifespan and healthspan extension in C. elegans via CF2.
