# Supplementary material for: Human Umbilical Cord Plasma Metabolomics Uncover Potential Metabolites for Combating Aging
Source: Aging Cell. 2025 Nov 26;25(1):e70295. doi: 10.1111/acel.70295 (PMC12740092; doi:10.1111/acel.70295)
Supplement: Supplementary file 2 — Appendix S2: acel70295‐sup‐0002‐AppendixS2.docx. [file ACEL-25-e70295-s009.docx]

**Supplementary information**

**Table S1 Demographic and clinical characteristics of**

**human umbilical cord plasma samples​**

| **Characteristics** | **Cohort 1**  **N=30** | **Cohort 2**  **N=30** |
| --- | --- | --- |
| Maternal age at birth (years) | 29.9 ± 2.3 | 30.8 ± 3.3 |
| Pre-pregnancy BMI (kg m^-2^) | 21.2 ± 2.5 | 21.3 ± 3.0 |
| Late pregnancy BMI (kg m^-2^) | 26.7 ± 3.4 | 26.9 ± 3.3 |
| ​​Neonatal BMI (kg m^-2^) | 13.1 ± 1.0 | 13.3 ± 1.1 |
| Female child (%) | 50 | 50 |

| **Characteristics** |  | | **Cohort 1** | |  | **Cohort 2** | | |
| --- | --- | --- | --- | --- | --- | --- | --- | --- |
|  | **Young**  **N=60** | **Midlife**  **N=60** | | **Elderly**  **N=60** | **Young**  **N=30** | | **Midlife**  **N=30** | **Elderly**  **N=30** |
| Female (%) | 50 | 50 | | 50 | 50 | | 50 | 50 |
| Age(years) | 22.73±2.02 | 45.79±2.77 | | 74.24±6.41 | 22.37±1.79 | | 47.27±4.53 | 69.65±4.49 |
| BMI（kg m^-2^) | 23.33±2.89 | 24.13±2.65 | | 23.46±2.61 | 23.53±3.47 | | 24.20±2.28 | 24.83±2.56 |

**Table S2 Demographic and clinical characteristics of adult peripheral blood plasma samples​**

**Table S11 Effect of CF1 on the lifespan of *C. elegans***

| **Treatment** | **Total number of nematodes** | **Mean lifespan**  **(days)** | **Maximum lifespan**  **(days)** | **Mean fold**  **increase (%)** |
| --- | --- | --- | --- | --- |
| H_2_O | 109 | 20.62 ± 5.44 | 29.45 ± 2.19 |  |
| 100μM CF1 | 109 | 23.48 ± 5.44*** | 32.18 ± 2.29* | 13.87 |

****P* < 0.05; *** *P* < 0.001 compared with control group**

**Table S12 Effect of CF2 on the lifespan of *C. elegans***

| **Treatment** | **Total number of nematodes** | **Mean lifespan**  **(days)** | **Maximum lifespan**  **(days)** | **Mean fold**  **increase (%)** |
| --- | --- | --- | --- | --- |
| H_2_O | 118 | 24.89 ± 5.37 | 34.83 ± 3.89 |  |
| 100μM CF2 | 120 | 27.42 ± 5.28*** | 38.25 ± 3.51* | 10.16 |

****P* < 0.05; ****P* < 0.001 compared with control group**

**Table S13 Primers for real-time polymerase chain reaction**

| **Gene name** | **Species** | **Forward** | **Reverse** |
| --- | --- | --- | --- |
| *p16* | human | CTTCCTCGGGTGCCGATAC | ACCCCTTCATTGCTACTCGAT |
| *p21* | human | TGTCCGTCAGAACCCATGC | AAAGTCGAAGTTCCATCGCTC |
| *IL6* | human | TTCTGCGCAGCTTTAAGGAG | AGGTGCCCATGCTACATTTG |
| *IL8* | human | ATGACTTCCAAGCTGGCCGTG | TGTGTTGGCGCAGTGTGGTC |
| *IL1α* | human | AGATGCCTGAGATACCCAAAACC | CCAAGCACACCCAGTAGTCT |
| *IL1β* | human | CTGTCCTGCGTGTTGAAAGA | TTGGGTAATTTTTGGGATCTACA |
| *CXCL1* | human | GCTGAACAGTGACAAATCCAAC | CTTCAGGAACAGCCACCAGT |
| *CXCL2* | human | CCCATGGTTAAGAAAATCATCG | CTTCAGGAACAGCCACCAAT |
| *GAPDH* | human | TCAACAGCAACTCCCACTCTTCCA | ACCCTGTTGCTGTAGCCGTATTCA |

**Supplementary Figure legends**

**Figure S1. Identification and functional analysis of potential pro-aging metabolites.** (A) Heatmap showing detailed intensity of all top differing metabolites across datasets, where each row corresponds to a metabolite and each column corresponds to a sample. Sample groups are marked at the bottom of the heatmap. (B) Pathway enrichment analysis showing differing pathways in different age groups with FDR < 0.05. Red circles denote significant differing pathways.

**Figure S2. Abundance pattern verification of candidate anti-aging metabolites.** (A–C) Quantitation of carnosine (A), taurocholic acid (B) and inosine (C) in plasma from independent cohort 2 (30 samples per group) using targeted mass spectrometry. (D–I) Quantitation of N1, N12–diacetylspermine (D), Kynurenine (E), Taurine (F), Hypoxanthine(G), Choline (H) and 1- methyladenosine (I) in plasma from the sub-cohort (10 samples per group). Statistical significance was determined via unpaired t test, with annotations: **p* < 0.05, ***p* < 0.01, ***p* < 0.001 (cord blood vs. adult groups).

**Figure S3. Validation of CF1 metabolites and associated enzyme expression across age cohorts.**

(A-E) The confirmation of five metabolites (Carnosine (A), Taurocholic acid (B), Inosine (C), L-Histidine (D), and N-Acetylneuraminic acid (E)) by standard compounds. Measured MS/MS spectral fragmentation profiles (top, in black) match chemical standards (bottom, in red). (F-I) Relative abundance of the metabolites and the expression level of corresponding metabolic enzymes in PBMC, including carnosine and its synthase *CARNS1*(F), N-Acetylneuraminic acid and its synthase *SIAE* (G), inosine and its synthase *NT5C* (H), along with L-Histidine and its methyltransferase *METTL6* (I). The dot plot shows the relative abundance of metabolites, with each dot representing the value for an individual sample, and the black line denoting the median relative abundance per age group. Violin plots depict the normalized expression levels of enzymes catalyzing metabolite-related reactions, across all PBMC samples in each age group. For each panel (F–I), the reaction formula links the metabolite to its corresponding catalytic enzyme (gene symbol highlighted in red), showing substrate-to-product transformations. Statistical significance: **p* < 0.05, ***p* < 0.01, ****p* < 0.001.

**Figure S4.** **Intracellular levels of target metabolites in human embryonic lung fibroblasts treated with CF1.** (A-E) Quantification analysis of intracellular level of taurocholic acid (A), carnosine (B), N-acetylneuraminic acid (C), L-histidine (D), and inosine (E) in MRC-5 cells using targeted mass spectrometry. Cells were pretreated with 100 μM CF1 for 12 h (A–C) or 6 h (D–E), with H₂O as the control. Intracellular levels are expressed as ng/mg protein. Statistical analysis was performed using an unpaired t-test; significance is denoted as **p* < 0.05, ***p* < 0.01, ****p* < 0.001.

**Figure S5. Attenuation of stress-induced senescence in human embryonic lung fibroblasts by CF2.** (A) Heatmap displaying the mean normalized abundances of metabolites composing CF2. (B-C) Representative images (B) and statistical analysis (C) of SA-β-gal staining in MRC-5 cells pretreated with or without CF2 and then exposed to 10 μM etoposide for 24h, followed by washing and continuous culture in complete medium for 5 days. Scale bars, 25 μm. More than 500 cells were quantified in each group. (D) qPCR analysis of senescence-associated genes in MRC-5 cells treated as described in B. (E-F) Representative images (E) and quantitative analysis (F) of SA-β-gal staining in MRC-5 cells pretreated with or without CF2, exposed to 200 μM H₂O₂ for 1 hour, and then cultured for 3 days. Scale bars, 25 μm. More than 500 cells were quantified in each group. (G) Heatmap analysis of senescence-associated genes in MRC-5 cells treated as described in E. **p* < 0.05, ***p* < 0.01, ****p* < 0.001. by unpaired t test. Values are mean ± SD.

**Figure S6. Significant lifespan and healthspan extension in *C.elegans* via CF2.** (A) Lifespan of worms treated with CF2 or vehicle (H_2_O). *p* value represents comparison with vehicle calculated using long rank test. ***p* < 0.01. (B) Representative intestinal autofluorescence and brightfield images (*n* = 20 images per group) of worms on days 12 and 16 of adulthood after being treated with CF2 or vehicle. The blue autofluorescence in the figure indicates the accumulation of lipofuscin. Scale bars, 300 µm. (C) The comparison of relative fluorescence intensity of intestinal autofluorescence in groups as indicated in B. (D) Body bending frequency on days 11 and 15 of adulthood in worms treated with CF2 or vehicle (*n* = 20). (E) Pharyngeal pumping frequency on days 8 and 12 of adulthood in worms treated with CF2 or vehicle (*n* = 20). (F) The body length of worms on days 8 and 12 was measured. (G) The total number of progenies from day1 to day 4 was summarized. (H) Survival for day 2 nematodes treated with CF2 or vehicle (H_2_O) was recorded after being exposed to 35°C heat shock for 7 h. For D-F, statistical significance was calculated by two-way analysis of variance (ANOVA) followed by Bonferroni tests. For G-H, statistical significance was calculated by unpaired t test. **p* < 0.05, ***p* < 0.01, ****p* < 0.001; n.s., not significant; Values are mean ± SEM.
